# Modified ZhuJing pill protects retinal pigment epithelium against oxidative stress-induced epithelial–mesenchymal transition through Nrf2-mediated Akt/GSK3β pathway

**DOI:** 10.3389/fphar.2025.1545731

**Published:** 2025-05-30

**Authors:** Ning Yang, Caijian Xiong, Siqi Feng, Mengqi Gao, Siqi Zhou, Qinyi Hui, Xin Zhou, Qingzi Jin, Yan Shao, Xinrong Xu

**Affiliations:** ^1^ Department of Opthalmology, Affiliated Hospital of Nanjing University of Chinese Medicine, Nanjing, China; ^2^ Department of Ophthalmology, Liyang Hospital of Chinese Medicine, Liyang, China

**Keywords:** age-related macular degeneration, Nrf2 pathway, epithelial–mesenchymal transition, Akt/GSK3β pathway, modified ZhuJing pill

## Abstract

**Background:**

The modified ZhuJing pill (mZJP) has been widely used in China as a classical prescription for treating retinal diseases for years. Our preliminary experiment showed that mZJP exerted an antioxidant effect in treating dry age-related macular degeneration (AMD). Nevertheless, the specific mechanism underpinning the impact of mZJP on dry AMD remains obscure.

**Methods:**

The chemical metabolites of mZJP were qualitatively analyzed using LC-Q-TOF-MS. Dry AMD model mice were used to assess the efficacy of mZJP through optical coherence tomography (OCT), fundus autofluorescence (FAF), and immunofluorescence. Epithelial–mesenchymal transition (EMT) in OxLDL-induced ARPE-19 cells was evaluated by monitoring cellular integrity and quantifying EMT-related markers. Cell migration capacity was determined *via* wound healing and transwell assays. To investigate molecular mechanisms, cells were transfected with Nrf2 siRNA and analyzed through Western blotting, immunofluorescence, and migration assays under Nrf2 inhibition.

**Results:**

A total of 113 major metabolites were identified in mZJP. Our findings revealed that mZJP alleviated retinal pathological alterations and inhibited EMT progression. Furthermore, mZJP upregulated Nrf2 and HO-1 expression levels while downregulating Akt and GSK-3β phosphorylation levels. Notably, the EMT-suppressing effect of mZJP was significantly attenuated upon Nrf2 silencing, as evidenced by enhanced cell migration, decreased epithelial marker expression (E-cadherin), increased mesenchymal marker expression (vimentin and α-SMA), suppression of the Nrf2 pathway, and activation of the Akt/GSK3β pathway.

**Conclusion:**

Our study suggested that RPE protection by mZJP against oxidative stress induced EMT through Nrf2 activation and inhibition of the Akt/GSK3β pathway. MZJP could be a potential candidate drug for the treatment of dry AMD.

## 1 Introduction

Age-related macular degeneration (AMD) ranks as the third prevalent cause of low vision on a global scale (Mitchell P et al., 2018; GBD 2019 Blindness and Vision Impairment Collaborators, 2021). It is a prevalent irrevocable sight-threatening disease that primarily affects the retina’s macular region in older individuals. Normally, two types of AMD are encountered in clinical practice: wet AMD and dry AMD. Currently, 90% of total AMD is untreatable dry AMD (Kosmidou et al., 2018). Unfortunately, effective prophylactic measures or treatments for dry AMD have not yet been developed. AMD will continue to be a major cause of vision impairment among aging populations (GBD 2019 Blindness and Vision Impairment Collaborators, 2021). Therefore, exploring efficient interventions that target older adults with dry AMD is clearly warranted.

In recent studies, many factors have been implicated in AMD etiology and pathogenesis, but degeneration or dysfunction in RPE with advancing age makes a significant contribution (Kaarniranta et al., 2020). RPE cells are hexagonal-shaped highly polarized cells, which are significant for homeostatic regulation between the Bruch membrane and photoreceptors (Tong et al., 2023; Lazzarini et al., 2022). There is evidence that constant exposure to oxidative stress (OS) and EMT are thought to be the pathological factors leading to RPE abnormalities in the progression of AMD (Shen et al., 2023). Accumulated OS on aging-related conditions increases the overproduction of reactive oxygen species (ROS) in mitochondria and may induce RPE cell damage or innate immune system activation, contributing to RPE apoptosis and AMD progression (Huang et al., 2022; Handa, 2012). In animal models, oxidative damage caused by ROS overproduction has been shown to induce AMD-like phenotypes (Tong et al., 2023). In addition, extensive studies have confirmed that RPE cells transdifferentiate into mesenchymal cells *via* EMT, which plays a pivotal role in AMD (Shu and Lovicu et al., 2017; Blasiak et al., 2021; Llorián-Salvador et al., 2022). In this progression, RPE cells lose their normal phenotypes, and the amount of expression of adhesion and tight junction proteins in RPE also declines. There is ample evidence to support that ROS is involved in the generation and progression of EMT (Giannoni et al., 2012). Many pathways that trigger EMT are promoted by ROS (Hyttinen et al., 2019). As proof, ROS has the ability to directly activate transforming growth factor beta 1 (TGF-β1) and trigger the course of EMT *via* the involvement of the PI3K/Akt/mTOR signaling pathways (Yuan et al., 2022). Given the complicated mechanisms linked with the interaction among RPE, EMT, and ROS, whether the antioxidant pathway is critically involved in regulating EMT-mediated dry AMD should be explored for the effective therapeutic application for dry AMD.

Over the past two decades, many traditional Chinese medicines and their bioactive metabolites comprising abundant antioxidants have been found to be safe and effective in treating AMD (Li et al., 2022). mZJP, a Chinese prescription for treating retinal disease, is composed of *Cuscuta chinensis* Lam. [Convolvulaceae; Cuscutae semen], *Plantago asiatica* L. [Plantaginaceae; Plantaginis semen], *Lycium barbarum* L. [Solanaceae; Lycii fructus], *Broussonetia × kazinoki* Siebold [Moraceae; Broussonetiae cortex], *Leonurus japonicus* Houtt. [Lamiaceae; Leonuri herba], *Chaenomeles speciosa* (Sweet) Nakai [Rosaceae; Chaenomelis fructus], *Panax notoginseng* (Burkill) F.H.Chen [Araliaceae; Notoginseng radix], *Schisandra chinensis* (Turcz.) Baill. [Schisandraceae; Schisandrae fructus], *placenta Hominis* [Hominidae; Placenta Hominis (dried)], and Mirabilitum [Mirabilitum; Natrii sulfas (crystalline)]. The active metabolites of these 10 botanical drugs are believed to maintain retinal homeostasis through multiple approaches and synergistic activities. Through years of clinical practice in China, mZJP has received much attention in treating retinal diseases such as AMD, central serous chorioretinopathy, and retinitis pigmentosa (Lei et al., 2018; Meng, 2019; Zhang and Duan, 2022). Our clinical research showed that mZJP can not only improve visual acuity, reduce fundus AF intensity, and decrease macular drusen area but also increase the plasma SOD and GSH-Px activities and decrease the MDA level in patients with dry AMD (Feng et al., 2024). *In vivo*, mZJP reduced the sediment area under RPE and inhibited the thickening of Bruch membrane (BrM) through turning on the Nrf2/Keap1 pathway and upregulating its target genes HO-1 and NQO-1(Ke et al., 2021). It means that mZJP can accelerate the removal of oxidative damage to protect the retina of mice with dry AMD. Additionally, it was proven that the active metabolites of apigenin, quercetin, and luteolin in the formula had protective effects on dry AMD model mice and oxidant-induced RPE cell damage (Chen et al., 2022; Zhang et al., 2020; Xu et al., 2016). *In vitro*, mZJP-medicated serum suppressed H_2_O_2_-induced EMT in ARPE-19 cells, which suggested that inhibiting the EMT which is induced by oxidative damage may be the mechanism of mZJP in the treatment of dry AMD (Shao et al., 2022). Moreover, using TMT proteomics technology in another research, we recently reported (Zhong et al., 2024) five main candidate target proteins of mZJP for treating AMD with “liver and kidney deficiency” being screened out, namely, CDH1, CDC42, PTPRK, CYCS, and PTPRG. Pathway enrichment analysis revealed that the main signaling pathways were the EMT and Wnt pathways. Given the performance of mZJP in treating dry AMD, more research is needed to figure out how the mechanism functions.

## 2 Materials and methods

### 2.1 Preparation of mZJP

All crude botanical drugs of mZJP were processed into Chinese decoction-free granules at Jiangyin Tianjiang Pharmaceutical Co., Ltd. (National Drug Approval Word: Z20050425, Batch No. 14092201), according to the Good Manufacturing Practice for Drugs to guarantee the quality. Further details regarding mZJP are shown in Supplementary Table S1.

### 2.2 Quality control of mZJP

#### 2.2.1 Chromatographic conditions

An Agilent SB C18 column (4.6 mm × 100 mm, 1.8 μm) was used to conduct the chromatographic separation. The mobile phase was 0.1% aqueous formic acid (A) and methanol (106007, Merck Chemicals, Shanghai, China): acetonitrile (34851, Merck Chemicals, Shanghai, China) = 1:1 (v/v, B), with a gradient elution. The gradient program is shown in Supplementary Table S2.

#### 2.2.2 Mass spectrometry conditions

Qualitative analysis of mZJP was achieved on an AB SCIEX Triple ToF 5600 (AB SCIEX, Foster City, CA) with an ESI source. The mass spectrometry parameters were as follows: TEM, 550°C; CUR, 20  psi; GS 1, 50  psi; and GS 2, 60  psi. The scanning mass range was m/z 100–1,200. Analysis was conducted in two modes (negative and positive). Data were acquired in the information-dependent acquisition (IDA) mode, which set the 10 highest peaks with a response signal intensity >100 cps for mass scanning. Each sample was injected three times to assess instrument precision.

### 2.3 Animal

C57/BL6J mice (aged 6 months, weighing 25–33 g) were procured from Speiford Beijing Biotechnology Co., Ltd. (Beijing, China). The production license was SCXK (Beijing) 2019–0010. The guidelines for animal care were followed during all animal experiments, and the experimental scheme was granted approval by the Experimental Animal Ethics Committee of the Nanjing University of Chinese Medicine (approval number: 2020DW-36). The animals fall into six groups randomly, with 15 mice per group. The aging control group mice were given an ordinary diet for 9°months. The remaining 75 mice, regarded as model mice, were simultaneously given a high-fat diet for 6°months, followed by 0.8% HQ (J&K Scientific, Shanghai, China) added to drinking water for the last trimester. Then, these mice were subsequently partitioned into four groups randomly: the positive drug control group (lutein beta carotene and zinc gluconate soft capsule (LSD), 0.004 g/kg (Sikang Bioengineering, Hubei, China)) and three doses of mZJP groups (1.44, 2.88, and 5.76 g/kg/day). After the experiment, the mice were sacrificed for tissue sample collection.

### 2.4 Optical coherence tomography (OCT)

The mice pupils were observed to be totally dilated after administering 1% tropicamide (Kanda Pharmaceutical, Japan). After being anesthetized by 1.25% Avertin at 0.2 mL/10 g (AIBI Bio-Technology, Nanjing, China), the mice were positioned on a platform which was handmade for mice and could be conveniently aligned to the eyes. The average retinal thickness of the mice was measured by OCT (Intalight VG200D, China) using the macular cube scanning mode (Zhang et al., 2020). Sodium chloride injection ((0.9%, 10 mL), Otsuka Pharmaceutical, China) was administered into the mice eyes twice during examination to prevent cornea dehydration and improve the image quality.

### 2.5 Fundus autofluorescence (FAF)

The confocal scanning laser ophthalmoscope (Heidelberg Engineering, Germany) was employed for acquiring FAF images. The preparation for FAF was consistent with that for OCT, including anesthesia and pupil dilation. Then, the viscoelastic substance (Viscoat, Alcon-Couvreur, Belgium) was used in the cornea covered with a coverslip to minimize corneal refraction. Next, a 488-nm laser was applied to stimulate fluorescence. During imaging using the FAF, the mice were held steady by an assistant. The images were captured utilizing the Android runtime mode for quantitative FAF analysis, and ImageJ (version 1.8.0, NIH, MD, United States) was used to calculate the mean gray level in mice FAF images.

### 2.6 Immunofluorescence staining (IF)

For RPE/choroidal flat mounts, the cornea was pierced with a syringe needle (1 mL) after enucleation, and then the eyes were fixed in the FAS eyeball fixative solution (G1109-100ML, ServiceBio) at room temperature for 2 h. Following this, the anterior segment, vitreous, neuroretina, and sclera were removed. The sample was divided radially into four parts from the head of the optic nerve to the border. Subsequently, the RPE/choroidal complex was mounted onto a microscope slide, with the RPE positioned at the uppermost part. The sample was incubated with primary antibodies (ZO-1) overnight (4°C), and the secondary antibody was used to incubate the specimen for 1 h. At last, the acquisition of fluorescent digital images was achieved on a Leica 6000 fluorescence microscope (Leica, Germany). For the *in vitro* part, cells were subjected to treatment with mZJP or OxLDL as a control, followed by a subsequent washing step using PBS. They were next dealt with the appropriate primary antibodies (ZO-1 and vimentin) and fluorescence-coupled secondary antibodies. Following that, the nuclei were counterstained with DAPI.

### 2.7 Preparation of mZJP drug-containing serum

Thirty Sprague–Dawley (SD) rats (production license: SCXK (Jing)-2019-0010) were picked at random to separate the mZJP group, LSD group (positive drug), and blank serum group, and then gavaged with mZJP (1.786 g/kg), LSD (0.032 g/kg), and double-distilled water (10.0 mL/kg) for 7°days. The rats were euthanized subsequently to a 12-h fasting period using 1.25% Avertin solution administered at a dosage of 20 mL/kg. The blood specimens were obtained from the abdominal aorta, allowed to clot at ambient temperature, and then centrifuged (3,000 rpm/min, 15 min) to collect mZJP drug-containing serum and double-distilled water (ddH_2_O) serum. The samples were heat inactivated (56°C, 30 min) and stored at −80°C for later research.

### 2.8 Cell culture and transfection

Adult retinal pigment epithelial cell line-19(ARPE-19) cells were procured from the Procell Life Science &Technology Co., Ltd. (Wuhan, China, Cat NO: CL-0026) and were incubated in DMEM with 10% FBS and 1% P/S in humidified air (5% CO_2_, 37°C). The cells were treated with mZJP at three concentrations (2.5%, 5%, and 10%) for 24 h and subsequently with OxLDL (25 μg/mL (AngYu Biotechnologies, Shanghai, China)) for 24 h to explore the effects of mZJP in the presence of oxidative injury. To test whether Nrf2 contributes to the OxLDL-induced ARPE-19 cells’ EMT, siRNA was used to inhibit Nrf2 expression. siNrf2 and its negative control were obtained from Hunan Fenghui Co., Ltd. (Hunan, China). The cells were transfected, which is in accordance with the manufacturer’s manual (Lipofectamine 2000, Invitrogen).

### 2.9 Migration assay

#### 2.9.1 Wound healing assay

Cells were incubated until 80%–90% confluence in a 6-well plate. A straight-line scratch was made by a pipette tip. After rinsing with PBS, the cells were treated in the absence or presence of mZJP (2.5%, 5%, and 10%) and OxLDL for 24 h. Microscope images were taken of the wound after 0 and 24 h. Ultimately, the migratory capacity was assessed by means of the wound healing ratio.

#### 2.9.2 Transwell assay

A 24-well transwell chamber containing an 8 µm pore polycarbonate membrane insert (Corning) was applied for cell mobility assay. Fifty thousand ARPE-19 cells in 100 μL medium were seeded in the upper chamber. In the lower chamber, medium including 10% FBS was added. After 48 h of culture, the migrated cells were flushed with PBS, fixed with paraformaldehyde (4%), and then stained with crystal violet (0.1%) for 20 min. Under the microscope, five fields on the crosshead were selected for each group, and an average number of perforated cells in the fixed field of each group was calculated after taking images under the 20× objective.

### 2.10 Western blot analysis

Total protein was collected from ARPE-19 cells and the RPE–choroidal–scleral complexes of mice. The protein concentrations were determined using the method of the BCA Protein Assay (P0010, Beyotime). Equal sample amounts were separated using SDS-PAGE, followed by its transfer onto PVDF membranes (ISEQ00010, Millipore) and blocking for 2 h with 5% skimmed milk before incubating with primary antibody overnight (4°C). Following rinsing with TBST (three times, 10 min/per), the membranes were incubated with secondary antibodies (2 h) and then washed with TBST again. The bands were detected using Tanon’s ECL and quantifying analysis on ImageJ software. The antibodies used were as follows: α-SMA (1:10,000, Abcam), Nrf2 (1:1,000, CST), HO-1 (1:2,000, Abcam), GSK-3β (1:1,000, Abcam), p-GSK-3β (1:1,000, CST), Akt (1:1,000, Abcam), p-Akt (1:500, Abcam), and GAPDH (1:10,000, Abcam).

### 2.11 Experimental replicates

*In vivo* experiments: three independent cohorts (biological replicates, n = 3) were used for each experimental condition. Tissue samples from each animal were analyzed in triplicate (technical replicates) for protein expression and histology.

*In vitro* experiments: ARPE-19 cells from three distinct passages (biological replicates, n = 3) were cultured under OxLDL challenge. Each experiment within a passage was performed in quadruplicate (technical replicates). Data shown represent three independent experiments.

### 2.12 Statistical analysis

Data were shown as means ± SD. GraphPad Prism version 9.5 (GraphPad Software, United States) was deployed for all analysis. One-way analysis of variance (ANOVA) and Dunnett’s test were used for multigroup comparisons. *P* < 0.05 was considered statistically significant. Individual experiments were performed in at least triplicate samples.

## 3 Results

### 3.1 Identification and analysis of the metabolites in mZJP

**FIGURE 1:**
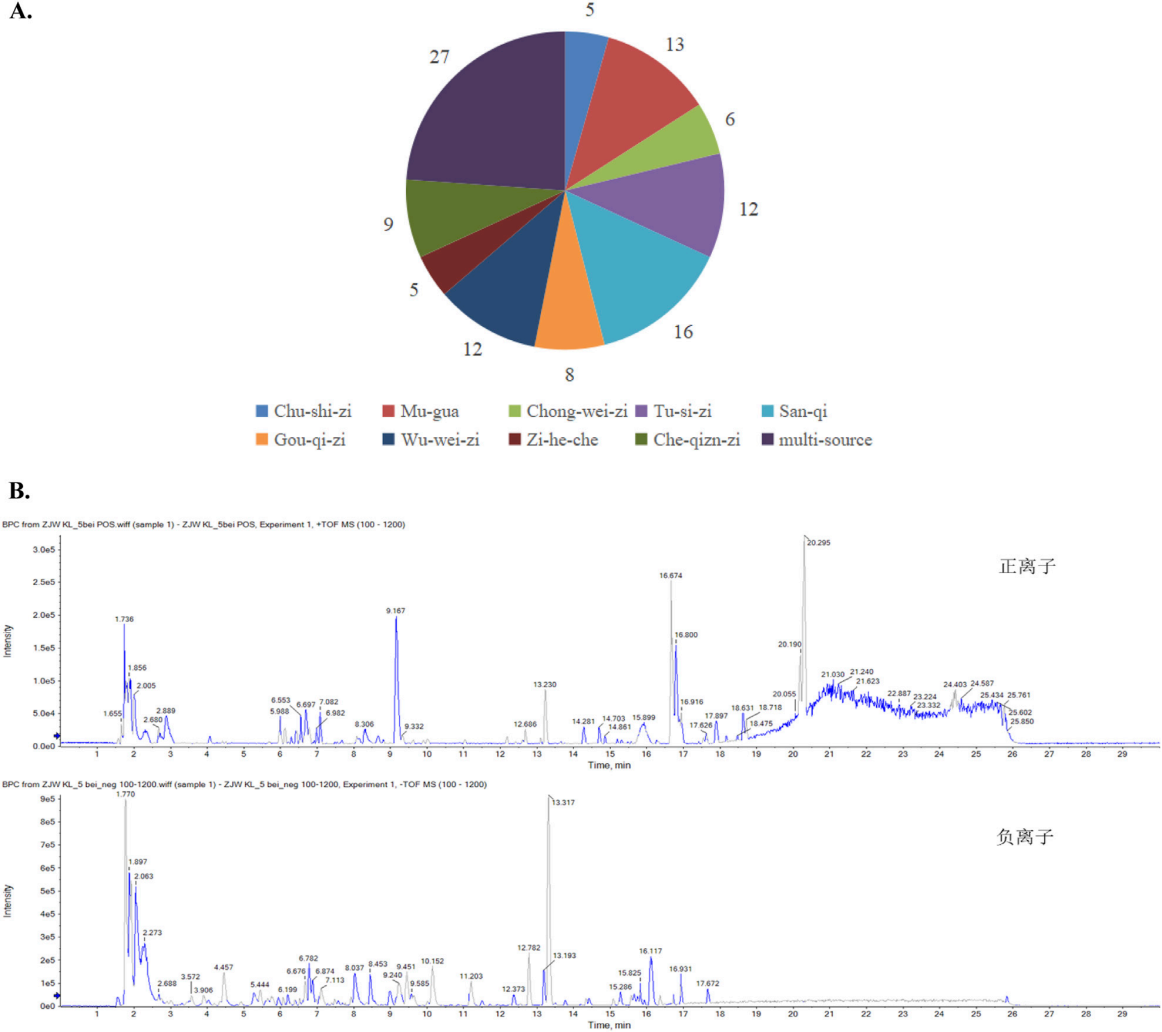
Qualitative analysis of the metabolites in mZJP granules. **(A)** Identification of the metabolites in mZJP. **(B)** BPC in positive (top) and negative (below) ion modes of mZJP granules (n = 3 per sample, technical replicates).

By contrasting the LC-Q-TOF-MS analysis with standards and references, the chemical metabolites of mZJP granules were determined. The ion peak in the two ion mode (positive and negative) was applied for the estimation of the relative molecular weight of the chemicals (Figure 1B). Furthermore, the chemical metabolites were qualitatively identified according to chromatographic retention time and MS/MS information (Supplementary Tables S3, S4). Finally, a total of 113 chemical metabolites were identified in mZJP granules (Figure 1A). As proof, five were unique to *Broussonetia × kazinoki* Siebold [Moraceae; Broussonetiae cortex] (in Chinese: Chu-shi-zi), 13 to *C. speciosa* (Sweet) Nakai [Rosaceae; Chaenomelis fructus] (in Chinese: Mu-gua), six to *L. japonicus* Houtt. [Lamiaceae; Leonuri herba] (in Chinese: Chong-wei-zi), 12 to *C. chinensis* Lam. [Convolvulaceae; Cuscutae semen] (in Chinese: Tu-si-zi), 16 to *P. notoginseng* (Burkill) F.H.Chen [Araliaceae; Notoginseng radix] (in Chinese: San-qi), eight to *L. barbarum* L. [Solanaceae; Lycii fructus] (in Chinese: Gou-qi-zi), 12 to *S. chinensis* (Turcz.) Baill. [Schisandraceae; Schisandrae fructus] (in Chinese: Wu-wei-zi), five to *placenta Hominis* [Hominidae; placenta Hominis (dried)] (in Chinese: Zi-he-che), and nine to *P. asiatica* L. [Plantaginaceae; Plantaginis semen] (in Chinese: Che-qian-zi), and 27 metabolites were from multiple sources. Further detailed information about qualitative and quantitative analyses of mZJP is presented in Supplementary Material. These results provide evidence of the quality control of the mZJP.

### 3.2 MZJP alleviated the retinal pathological changes in model mice

**FIGURE 2:**
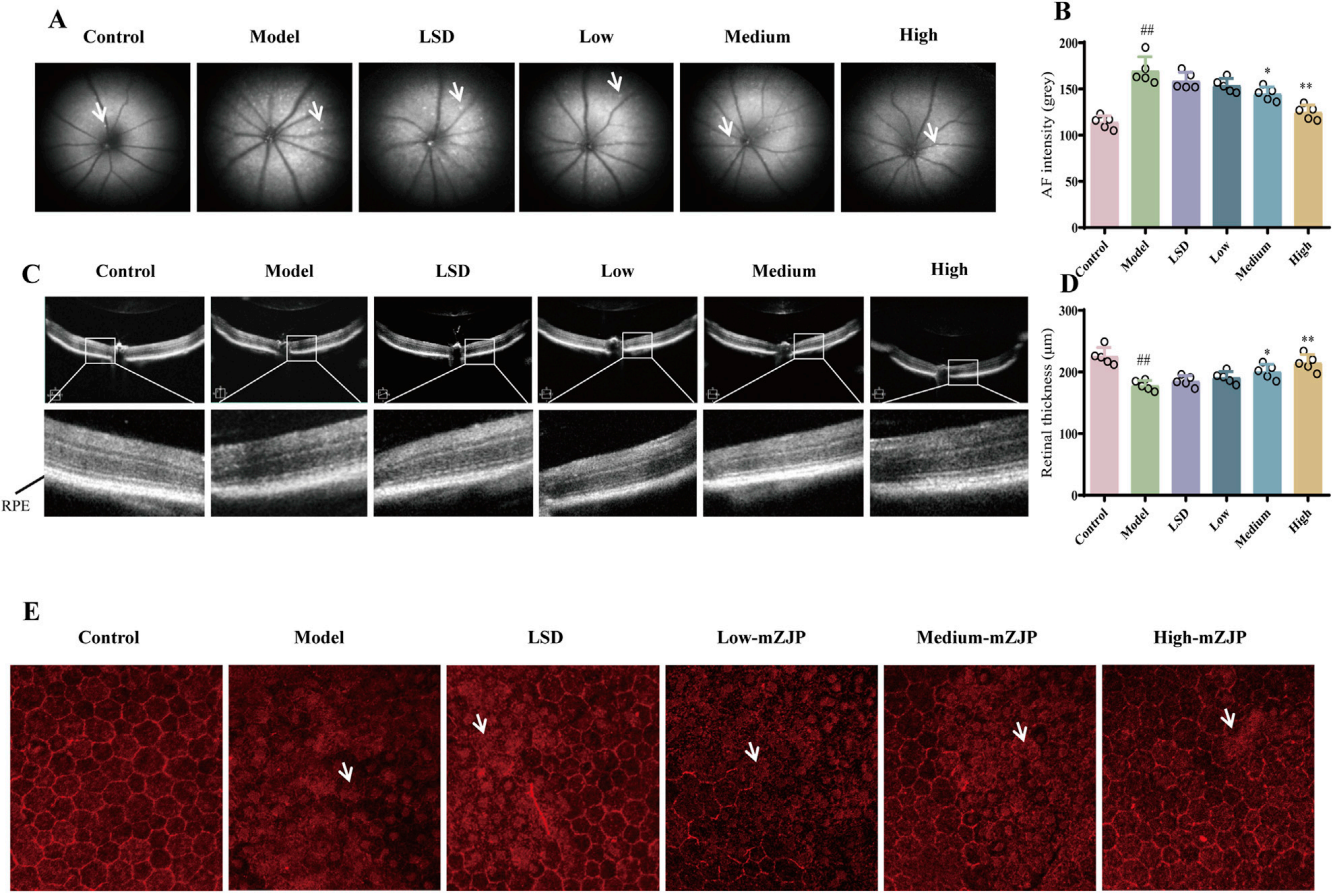
mZJP alleviated the retinal pathological changes in model mice. **(A)** Representative images of FAF. The white arrow points to the hyperfluorescence spots. **(B)** AF intensity to estimate the amounts of lipofuscin. LSD: positive drug control group. Mean ± SD, n = 5 mice per group, ^##^ *p* < 0.01; compared with the model group, ^*^ *p* < 0.05 and ^**^ *p* < 0.01. **(C)** Representative images of OCT. The black line points to the retinal pigment epithelium layer. **(D)** Retinal thickness to evaluate the effect of mZJP. Mean ± SD, n = 5 mice per group. ^##^ *P* < 0.01; compared with the model group, ^*^ *p* < 0.05 and ^**^ *p* < 0.01. **(E)** Immunofluorescence staining of ZO-1 on RPE/choroidal flat mounts. The white arrow points to the typical pathological changes. The images displayed are representative of the results from all mice in the same group (200×).

The FAF and OCT images helped monitor the pathological changes in RPE associated with AMD (Leuschen et al., 2013). In FAF, a direct correlation existed between fluorescence intensity and lipofuscin accumulation. The accumulation of RPE lipofuscin indicated hyperfluorescence, which was a characteristic of dry AMD (Schachar et al., 2013). The images indicated that FAF intensity was weakened following mZJP treatment in a dose-dependent manner. Moreover, the amount of subretinal deposition (hyperfluorescence spots) significantly increased in model mice, whereas mZJP treatment reversed the increase prominently (Figures 2A,B). In OCT, the cross-sectional images of the retina and choroid allowed clear visualization of the different retinal layers which were affected by AMD (Thomas et al., 2021). The retinal thickness of mice in the model group was substantially thinner than that of mice in the control group, and the photoreceptors and RPE became unremarkable. Conversely, the retinal layer structure became clear, and the structure of the outer retina was restorable to some extent in the mZJP-treated group (Figures 2C, D).

To further evaluate RPE integrity, we performed immunofluorescence staining of the EMT-related tight junction protein ZO-1 in RPE/choroid flat mounts. RPE health was assessed based on morphological features, including cell arrangement continuity and hexagonal tessellation patterns. As illustrated in Figure 2E, control group RPE cells exhibited a characteristic hexagonal honeycomb-like architecture with well-defined polygonal borders and uniform alignment. In stark contrast, model group mice displayed severe RPE disorganization, marked by disrupted cell integrity, irregular shapes, and loss of intercellular cohesion. mZJP treatment dose-dependently ameliorated these pathological changes. Whereas low-dose mZJP partially reduced cellular dispersion compared to that in the model group, medium-dose treatment restored modest hexagonal patterning. Notably, high-dose mZJP nearly normalized RPE morphology, achieving continuity and tessellation comparable to that in healthy controls. These findings demonstrate that mZJP effectively mitigates OS-induced RPE structural degeneration, with therapeutic efficacy correlating with dosage.

### 3.3 MZJP suppressed EMT and activated the Nrf2 pathway in model mice

**FIGURE 3:**
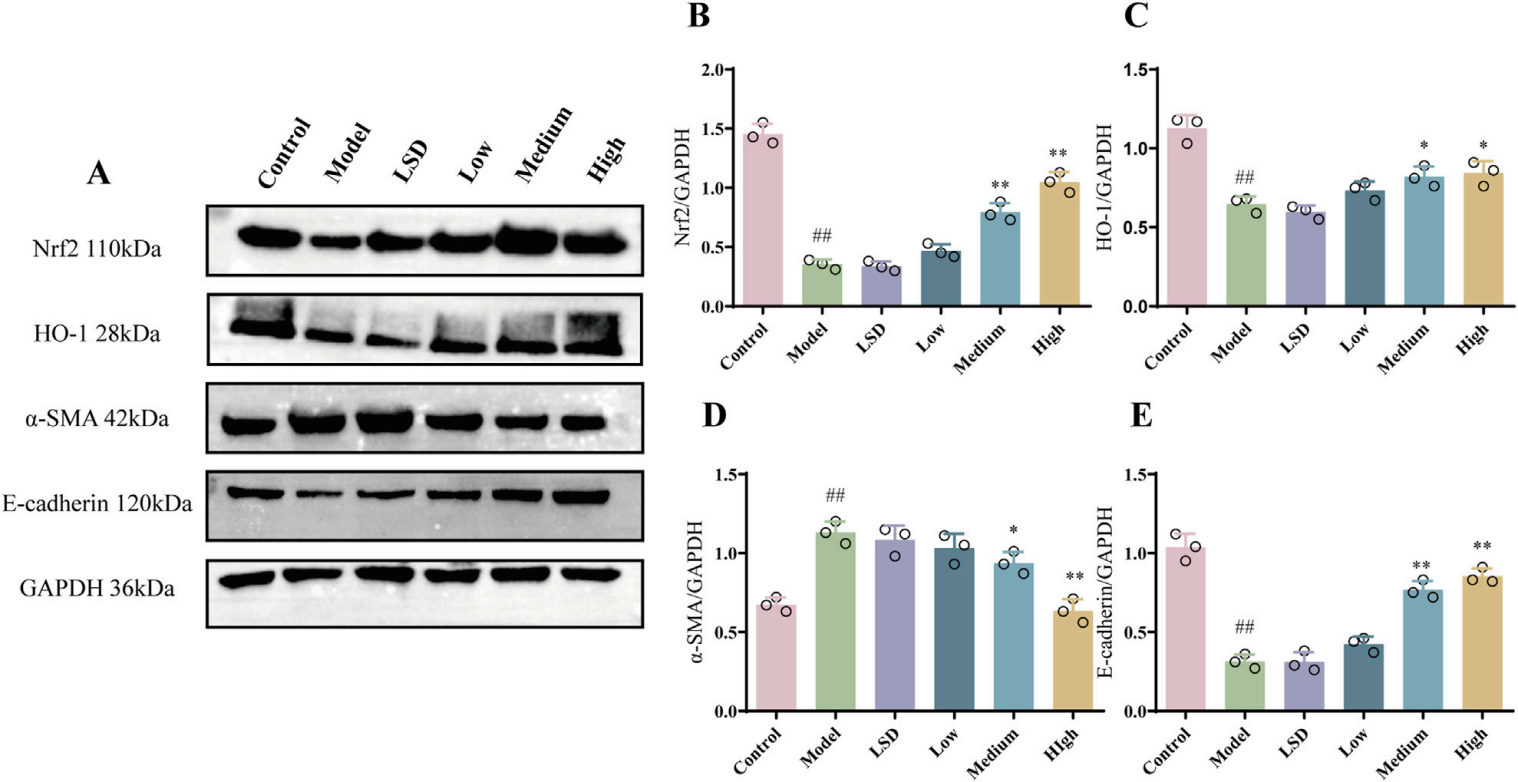
mZJP suppressed EMT and activated the Nrf2 pathway in model mice. **(A–E)** Protein levels of Nrf2, HO-1, α-SMA, and E-cadherin. GAPDH: internal control. Mean ± SD, n = 3 mice per group. Compared with the control group, ^##^ *p* < 0.01; compared with the model group, ^*^ *p* < 0.05 and ^**^ *p* < 0.01.

To determine the effect of mZJP on the EMT and Nrf2 pathway, Western blotting was used to examine related proteins. The α-SMA expression was attenuated, and the E-cadherin expression increased after treatment with mZJP when compared to that in the model group (Figures 3D,E). Moreover, as illustrated in Figures 3B, C, the expressions of Nrf2 and HO-1 decreased markedly in the model group, whereas mZJP treatment stimulated the expression in a dose-dependent manner, suggesting that mZJP may alleviate the abnormal retinal changes by suppressing EMT progression and activating the Nrf2 antioxidant pathway.

### 3.4 MZJP inhibited the Akt/GSK3β pathway in model mice

**FIGURE 4:**
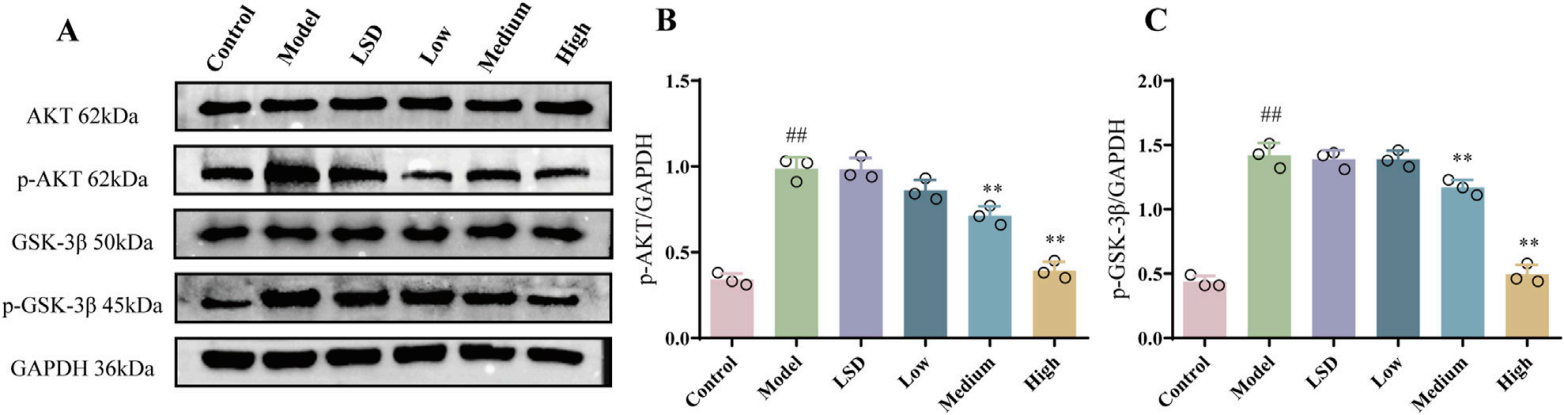
mZJP inhibited the Akt/GSK-3β pathway in model mice. **(A–C)** Protein expression of Akt, p-Akt, GSK-3β, and p-GSK-3β. In B and C, mean ± SD, n = 3 mice per group. Compared with the control group, ^##^ *p* < 0.01; compared with the model group, ^**^ *p* < 0.01.

Akt/GSK3β is a vital signal pathway in cells, which has been verified to pertain to the Nrf2 and EMT pathway (Zhang et al., 2022; Ouyang et al., 2021). On account of this relationship, we attempted to understand whether the Akt/GSK3β pathway was involved in the protective effect of mZJP. Our Western blot results (Figure 4) further supported this hypothesis. We found that the expressions of p-Akt and p-GSK3β in the model group were considerably raised compared to the expressions of those in the control group (p < 0.01). Compared with the model group and the LSD group, the medium- and high-dose mZJP treatment suppressed the expression markedly (*p* < 0.01), which indicated that the Akt/GSK3β signaling pathway might fulfill its role in this process.

### 3.5 MZJP suppressed OxLDL-induced cell migration in ARPE-19 cells

**FIGURE 5:**
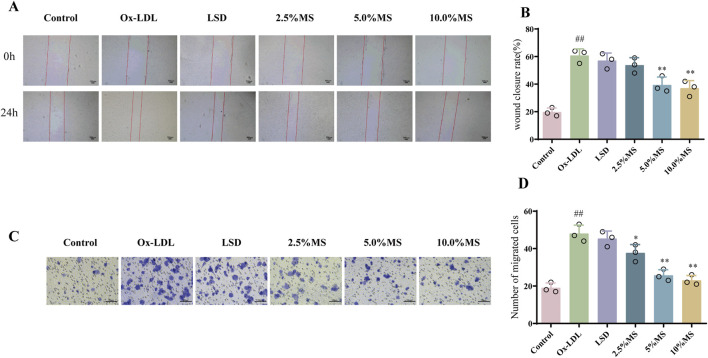
mZJP suppressed OxLDL-induced cell migration in ARPE-19 cells. **(A)** Images of wound healing assay for ARPE-19 cells migration in six groups were captured at 0 h and after 24 h (40×). **(B)** Wound closure was measured at 24 h after scratching. LSD: positive control group. Mean ± SD, n = 3. Compared with the control group, ^##^ *p* < 0.01; compared with the model group, ^**^ *p* < 0.01. **(C, D)** Transwell assay was used for estimating migration ability in OxLDL-treated ARPE-19 cells with or without mZJP after 48 h treatment (200×), and it was visualized using a graph. MS: medicine serum of mZJP. Mean ± SD, n = 3. Compared with the control group, ^##^ *p* < 0.01; compared with the model group, ^*^ *p* < 0.05, ^**^ *p* < 0.01.

As a biological program, EMT engages in retinal morphogenesis, which may lead to striking alterations in RPE cell migration (Boles et al., 2020). Therefore, migration assays were employed for exploring the effect of mZJP on OxLDL-induced ARPE-19 cells. As shown in Figure 5, OxLDL stimulation dramatically exhibited faster migration than the control group cells. On the contrary, it makes alterations after mZJP intervention, such as showed slower mobility and scratch closure. These results suggested that mZJP treatment can reduce the migration activity of OxLDL-induced ARPE-19 cells.

### 3.6 MZJP attenuation of OxLDL-induced EMT is associated with Akt/GSK3β-mediated Nrf2 activation

**FIGURE 6:**
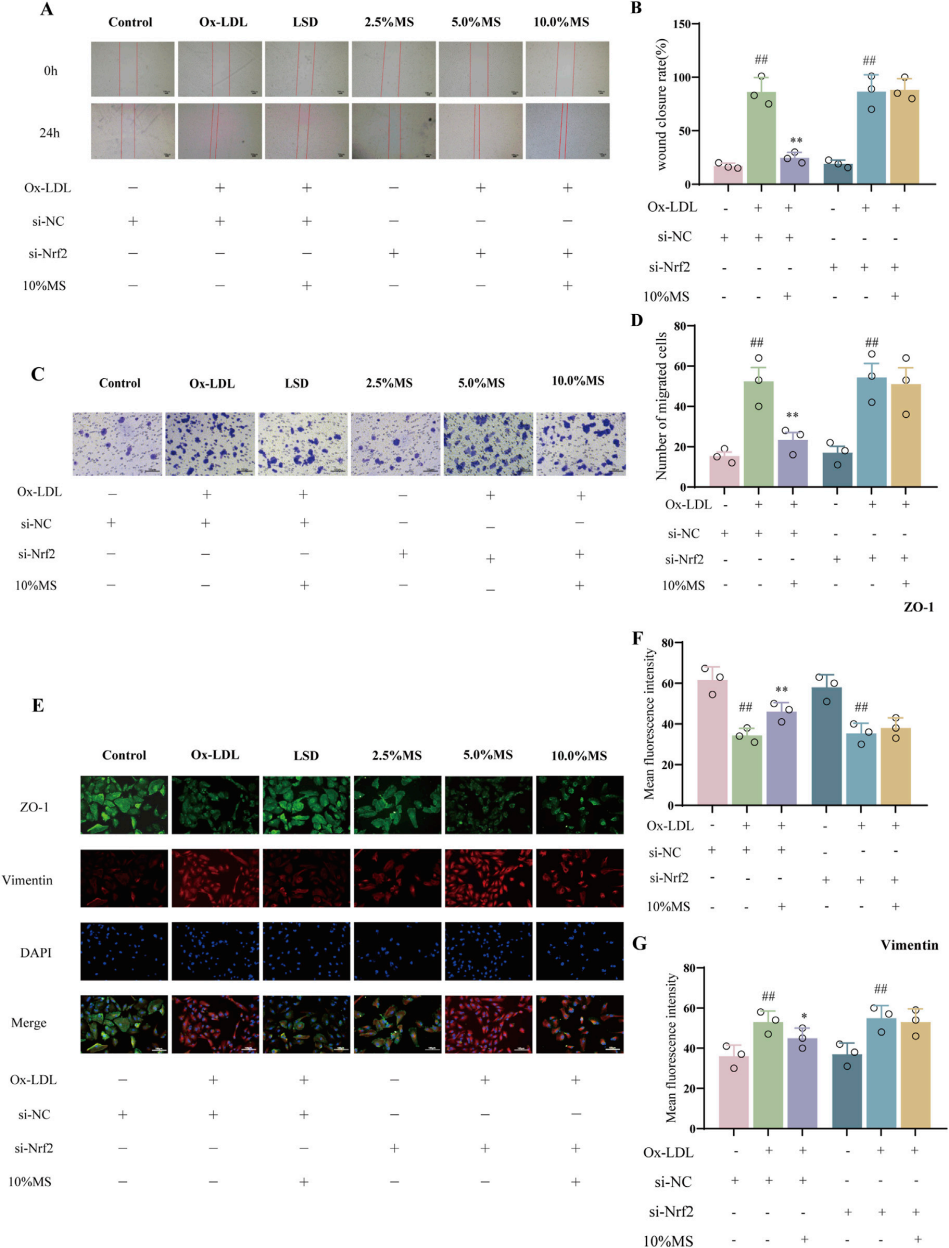
mZJP-attenuated OxLDL-induced EMT is associated with Akt/GSK3β-mediated Nrf2 activation (1). ARPE-19 cells were transfected with NC-siRNA or Nrf2-siRNA sequences, incubated with/without 10% mZJP for 24 h, and stimulated with OxLDL for 24 h **(A, B)**. Images of wound healing assay for ARPE-19 cell migration in the six groups were captured at 0 h and after 24 h (40×). Wound closure was measured at 24 h after scratching. Mean ± SD, n = 3. Compared with the control group, ^##^ *p* < 0.01; compared with the model group, ^**^ *P* < 0.01. **(C, D)** Transwell assay under Nrf2 silenced condition (200×). It was visualized using graph. Mean ± SD, n = 3. Compared with the control group, ^##^ *p* < 0.01; compared with the model group, ^**^ *p* < 0.01. **(E–G)** Immunofluorescence staining of ZO-1 (green) and vimentin (red) was detected by fluorescence microscopy. ImageJ software was used to analyze the fluorescence intensity of ZO-1 and vimentin (200×). MS: mZJP. Mean ± SD, n = 3. Compared with the control group, ^##^ *p* < 0.01; compared with the model group, ^**^ *p* < 0.01.

**FIGURE 7:**
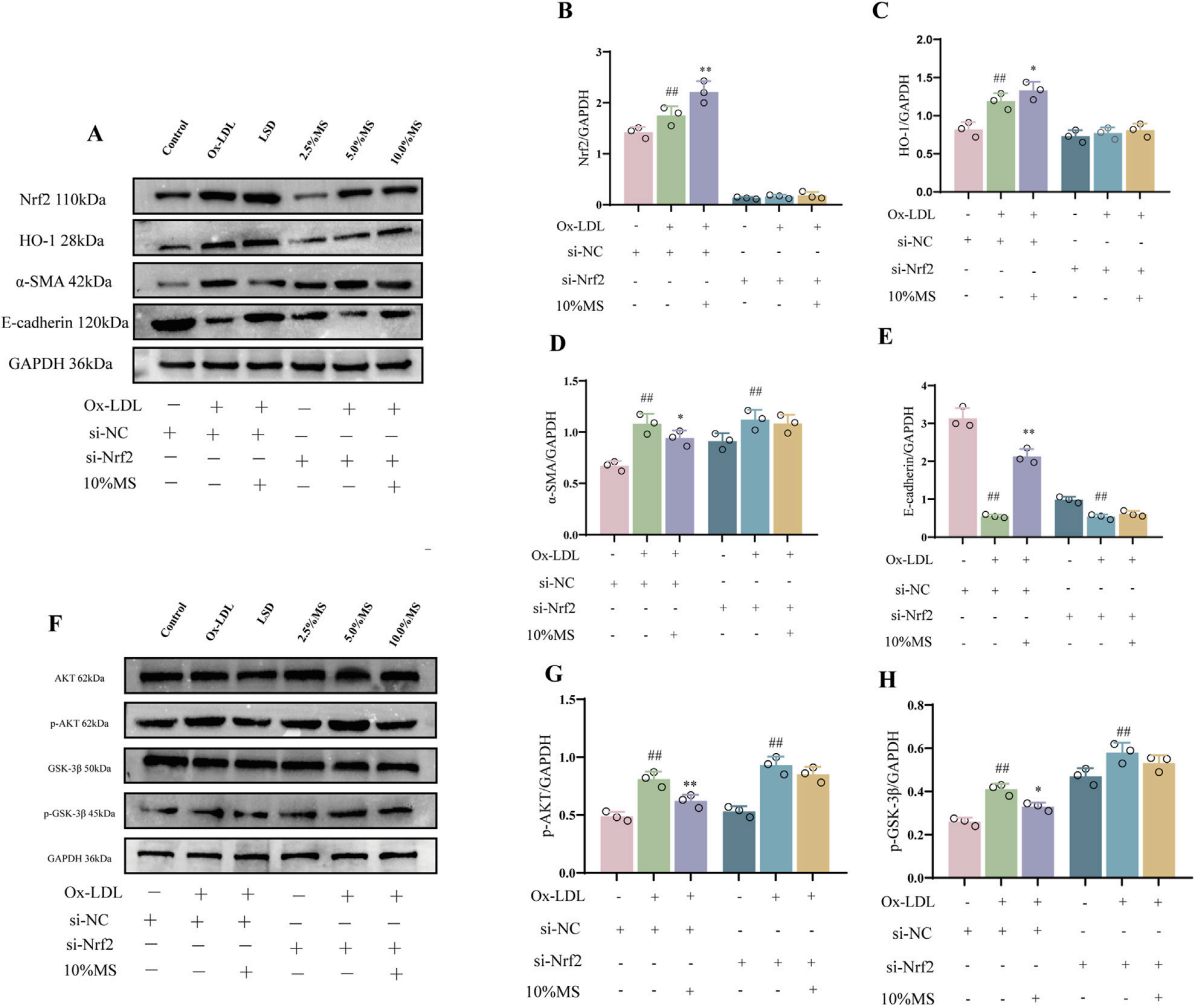
mZJP-attenuated OxLDL-induced EMT is associated with Akt/GSK3β-mediated Nrf2 activation (2). ARPE-19 cells were transfected with Nrf2 siRNA (+) or siRNA (−) for 24 h, incubated with/without 10% mZJP for 24 h, and stimulated with OxLDL for 24 h **(A–E)** Nrf2- and EMT-related proteins (Nrf2, HO-1, α-SMA, and E-cadherin) were used for Western blot analysis. **(F–H)** Expression level of Akt/GSK-3β pathway-related proteins. In **(B–E, G–H)**, mean ± SD, n = 3. Compared with the control group, ^##^ *p* < 0.01; compared with the model group, ^*^ *p* < 0.05 and ^**^ *p* < 0.01.

To elucidate if mZJP suppressed OxLDL-induced EMT by activating Nrf2, Nrf2 siRNA was utilized in OxLDL-induced ARPE-19 cells to block Nrf2 expression. As shown in Figures 6A–D, OxLDL promoted the migratory ability of ARPE-19 cells, which were further enhanced slightly in the presence of Nrf2 siRNA. Similarly, the suppressive action of mZJP on the migratory ability of OxLDL-induced ARPE-19 cells was found to be crippled in the presence of Nrf2 siRNA, but there was no statistical significance. Then, immunofluorescence (IF) was used to examine the EMT-related molecules in OxLDL-induced ARPE-19 cells. mZJP attenuated morphological alterations induced by OxLDL compared to that in the model group. However, Nrf2 siRNA reversed the inhibition of EMT by mZJP, which was embodied in ZO-1 and vimentin levels (Figures 6F,G). In line with IF findings, Western blot results of EMT-related proteins showed that when Nrf2 was knocked down, OxLDL-induced EMT progressed, the expression levels of α-SMA upregulated, and E-cadherin decreased (Figures 7D,E). These results suggested that OxLDL could induce EMT in human ARPE-19 cells and that Nrf2 was engaged in the process of mZJP ameliorating the progression of EMT. As was observed, Western blot showed that treatment with OxLDL increased Nrf2 and HO-1 expressions before Nrf2 transfection, which illustrated the existence of OS. However, after inhibiting Nrf2 expression, the protective effect of mZJP on OxLDL-induced EMT was eliminated, which was reflected by a marked decrease of Nrf2 and HO-1 (Figures 7B,C). At last, given that the Akt/GSK-3β pathway plays a significant mediatory role in Nrf2 and EMT pathways, we further assessed whether the effect of mZJP on the Akt/GSK3β pathway was mediated by Nrf2. The expression levels of related proteins associated with the Akt/GSK3β pathway on the condition of siNRF2 were examined by Western blot. The results revealed that OxLDL treatment significantly increased the expression of phosphorylated Akt and GSK3β on the condition of si-NC, which could be reversed by pretreatment with mZJP. However, when Nrf2 was knocked down, the effect of mZJP on the downregulation of Akt and GSK-3β was weakened (Figures 7G,H). Taken together, we suggest that the ability of mZJP to inhibit OxLDL-induced EMT depends on the Nrf2-mediated Akt/GSK3β pathway.

## 4 Discussion

It was reported that RPE suffer OS and EMT, which is thought to cause AMD (Yang et al., 2023; Shen et al., 2023). Therefore, inhibiting the progression of EMT, which is caused by oxidative damage, may be a key approach to treating dry AMD. Our study verified for the first time that mZJP has the ability to attenuate the retinal pathological changes and EMT progression in oxidative damage-induced model *in vivo* and *in vitro* by activating the Nrf2 and Akt/GSK3β pathway, among which the regulation of Nrf2 plays a key role.

Oxidative damage caused by aging, smoking, and dyslipidemias has long been a vital risk factor for dry AMD, which induces retinal changes in the degeneration of RPE, lipofuscin deposition, and drusen formation (Stahl, 2020; Ruan et al., 2021). Based on this, research workers attempted to study the pathogenesis, treatment, and prevention of dry AMD using animal models that mimicked the pathological features of AMD in humans. Recently, feeding aging mice with a high-fat diet and HQ mixed in water has been considered as a reliable model of dry AMD, which can reproduce various pathological features of dry AMD (Armento et al., 2025; Zheng et al., 2023; Cao et al., 2023; Sterling et al., 2022). Indeed, we have observed similar dry-AMD retinal changes in our model mice likewise. The OCT and FAF examinations revealed that compared with the control group, the model group obviously had the pathological features of dry AMD (drusen, focal atrophy of photoreceptors and RPE, and lipofuscin accumulation). It is notable that these pathological features were alleviated in the mZJP group, indicating that mZJP had protective effects on the retina of the model mice.

In recent years, EMT has been recognized as having an emerging role in dry AMD. It is a dynamic and transitional process allowing RPE cells to fight against OS for survival, but it causes the loss of normal epithelial phenotype and function (Ghosh et al., 2018). EMT is related to cell invasion and cell migration (González-Chavarría et al., 2018), which is a process that dysfunctional RPE cells would experience. Specific indications in our research studies were as follows: acceleration of cells migration and wound closure in the OxLDL-induced ARPE-19 model group. In addition, the decreased expressions of epithelial markers (E-cadherin and ZO-1) and the increased expression of mesenchymal markers (N-cadherin and vimentin) were observed in the model group. It is noteworthy that mZJP reversed these changes, which suggested that mZJP inhibited oxidative damage-induced EMT.

Nrf2, a critical transcription factor regulating cytoprotective genes, plays pivotal roles in counteracting inflammation, OS, and EMT (Wang et al., 2022). Accumulating evidence demonstrates that Nrf2 activation inhibits EMT progression in diabetic retinopathy and proliferative vitreoretinopathy models (Ouyang et al., 2022; Parikh et al., 2022). Furthermore, Nrf2-mediated upregulation of downstream targets HO-1 and NQO1 has been shown to suppress EMT-driven pulmonary fibrosis (Zhang et al., 2018). Paradoxically, the recent work by Zhang M et al. revealed a context-dependent pro-metastatic role of Nrf2 in cervical cancer *via* Snail1 induction, highlighting its functional duality (Zhang et al., 2023). These contrasting observations prompted us to investigate whether Nrf2 serves as a key mediator of mZJP’s inhibitory effects on EMT in our AMD model. Our experimental approach involved two phases: first, we confirmed mZJP’s ability to upregulate Nrf2-related proteins *in vivo*. Second, we employed Nrf2-silenced (siRNA-transfected) ARPE-19 cells under OxLDL challenge to dissect Nrf2’s mechanistic contribution. Notably, Nrf2 knockdown exacerbated OxLDL-induced mesenchymal transition while attenuating mZJP’s anti-EMT efficacy, establishing its necessity for therapeutic effects. Emerging studies suggest that Akt/GSK3β signaling directly regulates Nrf2 activity through phosphorylation-dependent modulation of its stability and nuclear translocation (Li et al., 2024; He et al., 2019). Considering the pivotal role of GSK3β in EMT and its putative interaction with Nrf2 (Shin et al., 2019; Medici et al., 2011; Peinado et al., 2005), we further investigated the Akt/GSK3β signaling pathway. Although Nrf2 regulation primarily involves Keap1 or Akt/GSK3β pathways, we focused on the Akt/GSK3β pathway due to its multifaceted downstream effects (e.g., mTOR and FOXO modulation) (Pan et al., 2023; Tang et al., 2021). Direct Nrf2 targeting allowed us to isolate its specific contribution from broader Akt signaling outputs, which could otherwise confound interpretation through parallel pathways affecting apoptosis, autophagy, and metabolism (Qu et al., 2023; Matta and Kumar, 2015). Our data reveal that mZJP inhibits EMT *via* Nrf2-dependent suppression of the Akt/GSK3β pathway. Nrf2 silencing significantly altered phosphorylated protein expression within this pathway while diminishing mZJP’s protective effects. Mechanistically, Nrf2 activation counteracts OS-induced EMT by attenuating Akt/GSK3β signaling, with mZJP exerting its therapeutic action through Nrf2 potentiation. Collectively, these findings position Nrf2 as a critical molecular target for mZJP in AMD intervention.

Our study supported the use of mZJP as a new treatment option and a potential drug candidate for dry AMD. Nevertheless, this study had some limitations. Whereas we focused on the Nrf2 and EMT pathways due to their established relevance to dry AMD, OS involves complex crosstalk among multiple signaling pathways, and the multi-target nature of traditional Chinese medicine metabolites suggests broader mechanistic interactions. Although our findings indicated that mZJP attenuated OS-induced EMT *via* Nrf2-mediated Akt/GSK3β activation, they fall short of comprehensively delineating causal relationships or spatial dynamics within the pathway. Furthermore, the protective effects of mZJP on other OS-related pathways remain unexplored. Another limitation is the absence of *in vivo* pharmacokinetic data for mZJP components. These data are crucial to determine bioavailability and tissue distribution. Future studies will prioritize these analyses to fully characterize therapeutic mechanisms. Despite these limitations, our work verifies mZJP’s therapeutic potential in protecting RPE cells and highlights its role as a promising candidate for AMD treatment. Advancing this field will require systematic efforts to address these challenges, but our findings lay a critical foundation for further translational research exploration.

## Data Availability

The original contributions presented in the study are included in the article/Supplementary Material; further inquiries can be directed to the corresponding author.
