# Supplementary material for: Modified ZhuJing pill protects retinal pigment epithelium against oxidative stress-induced epithelial–mesenchymal transition through Nrf2-mediated Akt/GSK3β pathway
Source: Front Pharmacol. 2025 May 30;16:1545731. doi: 10.3389/fphar.2025.1545731 (PMC12162958; doi:10.3389/fphar.2025.1545731)
Supplement: Supplementary file 1 [file DataSheet1.docx]

Supplementary Material

**S1. Quality control of mZJP granules:**

**Table S1-1.** Information of mZJP granules .

| Species name | Batch No. |
| --- | --- |
| Cuscuta chinensis Lam. [Convolvulaceae; Cuscutae semen] | 18121953 |
| Plantago asiatica L. [Plantaginaceae; Plantaginis semen] | 18100733 |
| Lycium barbarum L. [Solanaceae; Lycii fructus] | 18120903 |
| Broussonetia × kazinoki Siebold [Moraceae; Broussonetiae cortex] | 18051443 |
| Leonurus japonicus Houtt. [Lamiaceae; Leonuri herba] | 18081613 |
| Chaenomeles speciosa (Sweet) Nakai [Rosaceae; Chaenomelis fructus] | 18100233 |
| Panax notoginseng (Burkill) F.H.Chen [Araliaceae; Notoginseng radix] | 18101613 |
| Schisandra chinensis (Turcz.) Baill. [Schisandraceae; Schisandrae fructus] | 18101553 |
| Placenta Hominis [Hominidae; Placenta Hominis (dried)] | 18081793 |
| Mirabilitum [Mirabilitum; Natrii sulfas (crystalline)] | 18100583 |

The plants name of this formula has been checked with <http://mpns.kew.org>.

**Table S 1-2.** Liquid chromatography gradient method (Qualitative analysis).

| Time  (min) | Flow  (mL/min) | A  (%) | B  (%) |
| --- | --- | --- | --- |
| 0 | 0.50 | 90 | 10 |
| 3.5 | 0.50 | 85 | 15 |
| 5.0 | 0.50 | 70 | 30 |
| 9.0 | 0.50 | 65 | 35 |
| 11.0 | 0.50 | 50 | 50 |
| 12.0 | 0.50 | 40 | 60 |
| 14.0 | 0.50 | 30 | 70 |
| 19.0 | 0.50 | 10 | 90 |
| 24.0 | 0.60 | 10 | 90 |
| 24.5 | 0.60 | 90 | 10 |
| 30.0 | 0.60 | 90 | 10 |

S1. 1 Sample Preparation

S1.1.1 Mixed Standard Solution (Qualitative)

10 μL aliquots of 100 μg/mL stock solutions (betaine, stachydrine, succinic acid, chlorogenic acid, acteoside, rutin, hyperoside, isoacteoside, ginsenoside Re, Rg1, Rg2, schisandrol A, ginsenoside Rd, progesterone, and deoxyschizandrin) were mixed with 87 μL 50% methanol to prepare a mixed standard solution (1.50 μg/mL for each compound). After vortex mixing, 5 μL supernatant was analyzed by LC-Q-TOF/MS.

S1.1.2 Calibration Curve Working Solutions (Quantitative)

Mixed standard solutions were prepared in 50% methanol at the following concentrations: 2500 ng/mL (succinic acid, betaine, stachydrine, ginsenoside Re, Rg2, Rd, hyperoside), 1850 ng/mL (chlorogenic acid), 500 ng/mL (rutin), 250 ng/mL (schisandrol A), 10 ng/mL (progesterone), 8 ng/mL (deoxyschizandrin). Calibration curve working solution ① (200 μL) was diluted with 800 μL 50% methanol (vortex-mixed), followed by serial dilution to prepare a concentration series (stored at 4°C). For analysis, 90 μL of each working solution was spiked with 10 μL internal standard (IS), vortex-mixed, and 5 μL supernatant was analyzed by LC-MS/MS.

S1.1.3  Internal Standard (IS) Working Solution (Quantitative)

Phenacetin, tinidazole, theophylline, and chlorzoxazone were weighed accurately, dissolved in 50% methanol, and prepared to 1 mg/mL stock solutions. IS working solutions were prepared by diluting stocks to: 200 ng/mL (phenacetin), 250 ng/mL (tinidazole), 1000 ng/mL (theophylline), 100 ng/mL (chlorzoxazone). Solutions were stored at 4°C.

S1.1.4 Preparation of mZJP (granules) sample

Qualitative analysis: 0.1 g mZJP particles were dissolved in 0.75 mL ultrapure water, sonicated (30 min, room temperature), mixed with 0.75 mL methanol, and sonicated again (10 min). The solution (1 mL) was centrifuged (10 min, 12000 rpm, 4°C), and 400 μL supernatant was diluted with 600 μL 50% methanol (vortex-mixed), followed by centrifugation (10 min, 12000 rpm, 4°C) to obtain Sample ① (5× dilution). Sample ① (200 μL) was further diluted with 600 μL 50% methanol to prepare Sample ② (20× dilution). Both samples (5 μL each) were analyzed by LC-Q-TOF/MS.

Quantitative analysis: Sample ② (200 μL) was diluted with 800 μL 50% methanol to prepare Sample ③ (100× dilution). Separately, 50 μL of Sample ② was mixed with 700 μL 50% methanol to prepare Sample ④ (300× dilution). Samples ③ and ④ (90 μL each) were spiked with 10 μL internal standard, vortex-mixed, and 5 μL supernatant was analyzed by LC-MS/MS.

**Table S1-3.** Identification of chemical compounds within mZJP granules by LC-Q-TOF-MS.

| NO. | Compounds | Molecular Formula | Retention time(Rt) | Exact molecular weight | Adduct ion | Other adduct ion | Theoretical molecular weight | | Measured molecular weight | Error | Intensity | Source |
| --- | --- | --- | --- | --- | --- | --- | --- | --- | --- | --- | --- | --- |
|  |  |  | (min) | (Da) |  |  | (Da) | (Da) | | （ppm） |  |  |
| 1 | Argininic acid | C6H14N4O2 | 1.66 | 174.1117 | +H | -H | 175.119 | 175.1188 | | -0.9 | 135135 | Tusizi |
| 2 | Mannose | C6H14O6 | 1.82 | 182.0790 | +H | -H | 183.0863 | 183.0858 | | -2.7 | 14514 | Mugua |
| 3 | Betaine* | C5H12NO2+ | 1.82 | 118.0868 |  |  | 118.0868 | 118.08669 | | -0.9 | 420600 | Gouqizi |
| 4 | Trigonelline | C7H7NO2 | 1.83 | 137.0477 | +H |  | 138.055 | 138.055 | | 0.2 | 85519 | Gouqizi |
| 5 | Proline | C5H9NO2 | 1.85 | 115.0633 | +H |  | 116.0706 | 116.0708 | | 1.4 | 127236 | Gouqizi |
| 6 | Quinic acid | C7H12O6 | 1.88 | 192.0634 | -H | +H | 191.0561 | 191.0566 | | 2.3 | 2116350 | Mugua |
| 7 | Stachydrine hydrochloride* | C7H14NO2+ | 1.9 | 144.1025 |  |  | 144.1025 | 144.1026 | | 0.8 | 425363 | Chongweizi |
| 8 | Glucose | C6H12O6 | 1.9 | 180.0634 | -H |  | 179.0561 | 179.0561 | | -0.1 | 65456 | Tusizi |
| 9 | [D-mannitol](https://pubchem.ncbi.nlm.nih.gov/compound/6251) | C6H14O6 | 1.91 | 182.079 | -H | +H | 181.0718 | 181.0721 | | 1.6 | 25120 | Mugua |
| 10 | Ascorbyl Glucoside | C12H18O11 | 1.92 | 338.0849 | -H | +H | 337.0776 | 337.0779 | | 0.9 | 2074350 | Gouqizi |
| 11 | β-oxoacteoside | C29H34O16 | 1.92 | 638.1847 | -H |  | 637.1774 | 637.1811 | | 5.7 | 23279 | Cheqianzi |
| 12 | Vitamin C | C6H8O6 | 1.94 | 176.0321 | +H |  | 177.0394 | 177.0392 | | -0.7 | 50186 | Gouqizi |
| 13 | Isochlorogenic acid A/B/C | C25H24O12 | 1.97 | 516.1268 | -H |  | 515.1195 | | 515.1242 | 9.1 | 48139 | Tusizi |
| 14 | Shikimic acid | C7H10O5 | 1.98 | 174.0528 | -H |  | 173.0456 | | 173.046 | 2.6 | 118954 | Mugua |
| 15 | fumaric acid | C4H4O4 | 2 | 116.011 | -H |  | 115.0037 | | 115.0047 | 8.4 | 95863 | Chongweizi, Mugua, Gouqizi |
| 16 | Malic Acid | C4H6O5 | 2 | 134.0215 | -H |  | 133.0143 | | 133.0152 | 7.3 | 366710 | Mugua, Gouqizi |
| 17 | Hypoxanthine* | C5H4N4O | 2.02 | 136.0385 | +H | -H | 137.0458 | | 137.0459 | 0.5 | 8773 | Ziheche |
| 18 | (Z)-Aconitic Acid | C6H6O6 | 2.04 | 174.0164 | -H |  | 173.0092 | | 173.0098 | 3.5 | 25244 | Mugua |
| 19 | Citric Acid | C6H8O7 | 2.06 | 192.027 | -H |  | 191.0197 | | 191.0204 | 3.5 | 1944070 | Mugua, Gouqizi |
| 20 | Uridine | C9H12N2O6 | 2.63 | 244.0695 | -H |  | 243.0623 | | 243.0625 | 0.8 | 36725 | Gouqizi |
| 21 | Guanosine* | C10H13N5O5 | 2.67 | 283.0917 | -H |  | 282.0844 | | 282.0844 | 0.1 | 19708 | Ziheche |
| 22 | Inosine* | C10H12N4O5 | 2.68 | 268.0808 | -H |  | 267.0735 | | 267.0738 | 1 | 14464 | Ziheche |
| 23 | l-isoleucine | C6H13NO2 | 2.78 | 131.0946 | +H | -H | 132.1019 | | 132.102 | 0.8 | 28388 | Ziheche, Gouqizi |
| 24 | Succinic acid* | C4H6O4 | 2.82 | 118.0266 | -H |  | 117.0193 | | 117.0204 | 8.7 | 49918 | Mugua |
| 25 | D-tert-Leucine | C6H13NO2 | 2.99 | 131.0946 | +H | -H | 132.1019 | | 132.102 | 1 | 47209 | Ziheche, Gouqizi |
| 26 | Gallic acid | C7H6O5 | 3.25 | 170.0215 | -H |  | 169.0143 | | 169.0149 | 3.6 | 31017 | Mugua |
| 27 | Sesaminol | C20H18O7 | 3.91 | 370.1053 | -H |  | 369.098 | | 369.1017 | 10 | 38155 | Tusizi |
| 28 | phenylalanine | C9H11NO2 | 3.91 | 165.079 | -H | +H | 164.0717 | | 164.0723 | 3.4 | 53134 | Gouqizi, Ziheche, Tusizi |
| 29 | [Nitidine](https://pubchem.ncbi.nlm.nih.gov/compound/4501) | C21H18NO4+ | 4.34 | 348.1236 |  |  | 348.1236 | | 348.1287 | 14.6 | 3623 | Chushizi |
| 30 | Geniposidic acid* | C16H22O10 | 4.46 | 374.1213 | -H | +H | 373.114 | | 373.1141 | 0.2 | 592725 | Cheqianzi |
| 31 | Neochlorogenic acid | C16H18O9 | 5.28 | 354.0951 | -H | +H | 353.0878 | | 353.0882 | 1.2 | 255705 | Tusizi |
| 32 | protocatechuic acid | C7H6O4 | 5.35 | 154.0266 | -H |  | 153.0193 | | 153.0201 | 5.1 | 127439 | Mugua, Gouqizi |
| 33 | Aucubin | C15H22O9 | 6.18 | 346.1264 | -H |  | 345.1191 | | 345.1194 | 0.7 | 22714 | Cheqianzi |
| 34 | Quercetin-3-O-robinoside | C27H30O17 | 6.65 | 626.1483 | -H | +H | 625.141 | | 625.1419 | 1.3 | 55525 | Tusizi， Sanqi |
| 35 | catechin | C15H14O6 | 6.68 | 290.079 | -H | +H | 289.0718 | | 289.0721 | 1.1 | 418600 | Mugua |
| 36 | Cryptochlorogenic acid* | C16H18O9 | 6.78 | 354.0951 | -H | +H | 353.0878 | | 353.0882 | 1.2 | 717874 | Tusizi, Mugua, Gouqizi |
| 37 | Chlorogenic acid* | C16H18O9 | 6.88 | 354.0951 | -H | +H | 353.0878 | | 353.0881 | 1 | 440855 | Tusizi, Mugua, Gouqizi |
| 38 | 4-Hydroxybenzoic acid | C7H6O3 | 6.91 | 138.0317 | -H |  | 137.0244 | | 137.0252 | 5.3 | 50935 | Mugua |
| 39 | quercetin-3-O-α-L-rhamnopyranosyl-(1→6)-β-D-galactopyranoside | C27H30O16 | 7.1 | 610.1534 | -H | +H | 609.1461 | | 609.1458 | -0.5 | 26160 | Tusizi |
| 40 | epicatechin* | C15H14O6 | 7.4 | 290.079 | -H | +H | 289.0718 | | 289.0719 | 0.5 | 64511 | Tusizi，Mugua |
| 41 | Caffeic acid* | C9H8O4 | 7.48 | 180.0423 | -H |  | 179.035 | | 179.0356 | 3.3 | 115096 | Tusizi, Mugua, Gouqizi |
| 42 | Phenylacetic acid | C8H8O2 | 7.49 | 136.0524 | -H |  | 135.0452 | | 135.046 | 5.9 | 21657 | Mugua |
| 43 | Daphnetin/Esculetin | C9H6O4 | 7.51 | 178.0266 | -H |  | 177.0193 | | 177.0199 | 3.1 | 17683 | Mugua |
| 44 | 8-epi-Loganic acid | C16H24O10 | 7.55 | 376.137 | -H | +H | 375.1297 | | 375.1309 | 3.4 | 14284 | Cheqianzi |
| 45 | Riboflavin | C17H20N4O6 | 7.55 | 376.1383 | +H | -H | 377.1456 | | 377.1459 | 0.9 | 33107 | Chongweizi, Gouqizi |
| 46 | Leonurine | C14H21N3O5 | 7.9 | 311.1481 | +H |  | 312.1554 | | 312.1551 | -0.9 | 13193 | Chongweizi |
| 47 | benzoic acid | C7H6O2 | 8.35 | 122.0368 | -H |  | 121.0295 | | 121.0304 | 7.1 | 62209 | Mugua |
| 48 | Quercetin-3-O-apose-(1→2)-galactoside | C26H28O16 | 8.45 | 596.1377 | -H | +H | 595.1305 | | 595.1299 | -0.9 | 595290 | Tusizi |
| 49 | 4-Hydroxycinnamic acid/ | C9H8O3 | 8.63 | 164.0473 | -H | +H | 163.0401 | | 163.0409 | 4.8 | 10890 | Chushizi, Mugua, Gouqizi |
|  | cis-Hydroxycinnamic acid |  |  |  |  |  |  |  |  |  |  |  |
| 50 | Naringenin-7-O-glucoside | C21H22O10 | 8.76 | 434.1213 | -H |  | 433.114 | | 433.1143 | 0.5 | 29929 | Mugua |
| 51 | Phenylacetaldehyde | C8H8O | 9.01 | 120.0575 | -H |  | 119.0502 | | 119.0513 | 8.9 | 38661 | Chongweizi |
| 52 | 5-Hydroxycinnamic acid/cis-Hydroxycinnamic acid | C9H8O3 | 9.01 | 164.0473 | -H | +H | 163.0401 | | 163.0409 | 4.8 | 65999 | Chushizi，Mugua, Gouqizi |
| 53 | Quercetin 3-O-robinobioside | C27H30O16 | 9.05 | 610.1534 | +H |  | 611.1607 | | 611.1613 | 1 | 23139 | Sanqi |
| 54 | Verbascoside* | C29H36O15 | 9.16 | 624.2054 | -H | +H | 625.2127 | | 625.2131 | 0.7 | 193365 | Cheqianzi, Chongweizi |
| 55 | Rutin | C27H30O16 | 9.17 | 610.1534 | -H | +H | 609.1461 | | 609.1467 | 1 | 146797 | Cheqianzi, Chongweizi, Mugua, Gouqizi |
| 56 | Hyperoside* | C21H20O12 | 9.45 | 464.0955 | -H | +H | 463.0882 | | 463.0883 | 0.3 | 660889 | Tusizi, Mugua, Gouqizi |
| 57 | Plantamajoside/Plantainoside D | C29 H36 O16 | 9.46 | 640.2003 | -H |  | 639.1931 | | 639.1926 | -0.7 | 24306 | Cheqianzi |
| 58 | Martynoside | C31H40O15 | 9.61 | 652.2367 | -H |  | 651.2294 | | 651.2301 | 1 | 11610 | Cheqianzi |
| 59 | Isoquercitrin | C21H20O12 | 9.64 | 464.0955 | -H |  | 463.0882 | | 463.0883 | 0.3 | 215237 | Tusizi |
| 60 | Scopoletin | C10H8O4 | 9.69 | 192.0423 | +H |  | 193.0495 | | 193.0494 | -0.5 | 6206 | Chushizi |
| 61 | ferulic acid* | C10H10O4 | 9.72 | 194.0579 | -H |  | 193.0506 | | 193.0514 | 3.8 | 24280 | Mugua, Gouqizi |
| 62 | Heteroclitin G | C22H24O7 | 9.76 | 400.1522 | +H |  | 401.1595 | | 401.1579 | -4.1 | 9994 | Wuweizi |
| 63 | Forsythoside B | C34H44O19 | 9.79 | 756.2477 | -H |  | 755.2404 | | 755.241 | 0.8 | 11549 | Cheqianzi |
| 64 | Calceolarioside A/B | C23H26O11 | 10 | 478.1475 | +H |  | 479.1548 | | 479.1549 | 0.2 | 6669 | Cheqianzi |
| 65 | Isoacteoside* | C29H36O15 | 10.14 | 624.2054 | -H | +H | 623.1981 | | 623.1974 | -1.2 | 810763 | Cheqianzi, Chongweizi |
| 66 | methyl sinapate | C12H14O5 | 10.33 | 238.0841 | +H |  | 239.0914 | | 239.0896 | -7.4 | 19399 | Tusizi |
| 67 | Kaempferol | C15H10O6 | 11.04 | 286.0477 | +H |  | 287.055 | | 287.0548 | -0.7 | 17778 | Gouqizi, Sanqi |
| 68 | Esculetin | C9H6O4 | 11.1 | 178.0266 | -H |  | 177.0193 | | 177.0198 | 2.7 | 1750 | Gouqizi |
| 69 | Astragalin/ Kaempferol-3-O-galactoside | C21H20O11 | 11.2 | 448.1006 | -H | +H | 447.0933 | | 447.0933 | 0 | 440665 | Tusizi，Cheqianzi, Mugua, |
| 70 | physcion diglucoside | C28H32O15 | 11.37 | 608.1741 | -H |  | 607.1668 | | 607.1671 | 0.4 | 15492 | Gouqizi |
| 71 | KaeMpferol 7-O-rhaMnoside | C21H20O10 | 11.51 | 432.1057 | +COOH |  | 477.1028 | | 477.1033 | 1.2 | 108231 | Sanqi |
| 72 | nepitrin | C22H22O12 | 11.51 | 478.1111 | -H | +H | 477.1039 | | 477.1034 | -1 | 108936 | Cheqianzi |
| 73 | Apigenin | C15H10O5 | 11.95 | 270.0528 | -H |  | 269.0456 | | 269.0474 | 6.7 | 396 | Chushizi |
| 74 | Azelaic acid | C9H16O4 | 12.37 | 188.1049 | -H |  | 187.0976 | | 187.0981 | 2.9 | 203872 | Chushizi |
| 75 | Cuscutoside D | C37H46O21 | 12.56 | 826.2532 | +Na |  | 849.2424 | | 849.2426 | 0.3 | 1129 | Tusizi |
| 76 | Notoginsenoside R1* | C47H80O18 | 12.78 | 932.5345 | +COOH | +Na | 977.5316 | | 977.5325 | 1 | 1006744 | Sanqi |
| 77 | Ginsenoside Re* | C48H82O18 | 13.19 | 946.5501 | +COOH | +Na,+H | 991.5472 | | 991.5482 | 0.9 | 672059 | Sanqi |
| 78 | Ginsenoside Mc | C41H70O12 | 13.31 | 754.4867 | +COOH |  | 799.4838 | | 799.4842 | 0.5 | 16082 | Sanqi |
| 79 | Ginsenoside Rf* | C42H72O14 | 13.32 | 800.4922 | +COOH | +Na,+H | 845.4893 | | 845.4901 | 1 | 3640449 | Sanqi |
| 80 | Tiliroside | C30H26O13 | 13.41 | 594.1373 | -H |  | 593.1301 | | 593.1295 | -1 | 18300 | Chongweizi |
| 81 | Quercetin* | C15H10O7 | 13.47 | 302.0427 | -H | +H | 301.0354 | | 301.0357 | 0.9 | 39417 | Cheqianzi, Chushizi, Tusizi, Chongweizi, Mugua, Gouqizi |
| 82 | Cuscutoside C | C26H28O12 | 14.4 | 532.1581 | +Na |  | 555.1473 | | 555.1463 | -1.9 | 1470 | Tusizi |
| 83 | Luteolin* | C15H10O6 | 14.43 | 286.0477 | -H | +H | 285.0405 | | 285.0409 | 1.6 | 138315 | Chushizi, Cheqianzi |
| 84 | Isorhamnetin | C16H12O7 | 14.65 | 316.0583 | -H | +H | 315.051 | | 315.0513 | 0.8 | 14397 | Tusizi, Gouqizi |
| 85 | Ginsenoside Rg1* | C42H72O14 | 15.02 | 800.4922 | +COOH | +Na,+H | 845.4893 | | 845.4897 | 0.5 | 7592 | Sanqi |
| 86 | Notoginsenoside R2/ | C41H70O13 | 15.29 | 770.4816 | +COOH | +Na | 815.4788 | | 815.4794 | 0.8 | 271789 | Sanqi |
|  | Ginsenoside F5 |  |  |  |  |  |  |  |  |  |  |  |
| 87 | Ginsenoside Rg2* | C42H72O13 | 15.58 | 784.4973 | +COOH | +Na,+H | 829.4944 | | 829.495 | 0.8 | 207856 | Sanqi |
| 88 | Ginsenoside F1* | C36H62O9 | 15.83 | 638.4394 | +COOH | +Na | 683.4365 | | 683.4369 | 0.6 | 252600 | Sanqi |
| 89 | Cuscuta acid C | C38H68O20 | 15.94 | 844.4304 | -H |  | 843.4231 | | 843.4226 | -0.6 | 120536 | Tusizi |
| 90 | Ginsenoside Rb1* | C54H92O23 | 16.11 | 1108.6029 | +COOH | +Na,+H | 1153.6001 | | 1153.599 | -1 | 265880 | Sanqi |
| 91 | Wogonin/Genkwanin | C16H12O5 | 16.44 | 284.0685 | -H |  | 283.0612 | | 283.0613 | 0.3 | 16988 | Chongweizi |
| 92 | Ginsenoside Rh1* | C36H62O9 | 16.66 | 638.4394 | +COOH | +Na | 683.4365 | | 683.4369 | 0.6 | 26887 | Sanqi |
| 93 | Wogonin/Genkwanin | C16H12O5 | 16.86 | 284.0685 | -H |  | 283.0612 | | 283.0614 | 0.8 | 9364 | Chongweizi |
| 94 | Veraguensin | C22H28O5 | 16.89 | 372.1937 | +H |  | 373.201 | | 373.2004 | -1.5 | 11666 | Wuweizi |
| 95 | Schisandrin* | C24H32O7 | 16.94 | 432.2148 | +H |  | 433.2221 | | 433.2217 | -1 | 148237 | Wuweizi |
| 96 | Gomisin J | C22H28O6 | 17.12 | 388.1886 | +H |  | 389.1959 | | 389.1961 | 0.7 | 11580 | Wuweizi |
| 97 | Ginsenoside RG5 | C42H70O12 | 17.55 | 766.4867 | +H |  | 767.494 | | 767.4943 | 0.4 | 39373 | Sanqi |
| 98 | Schisandrol B* | C23H28O7 | 17.63 | 416.1835 | +Na | +H | 439.1727 | | 439.1732 | 1 | 99108 | Wuweizi |
| 99 | Ginsenoside Rd* | C48H82O18 | 17.67 | 946.5501 | +COOH | +Na,+H | 991.5472 | | 991.5467 | -0.5 | 307617 | Sanqi |
| 100 | Angeloylgomisin H | C28H36O8 | 18.18 | 500.241 | +H |  | 501.2483 | | 501.2478 | -1 | 27831 | Wuweizi |
| 101 | Gomisin G | C30H32O9 | 18.34 | 536.2046 | +H |  | 537.2119 | | 537.2098 | -4 | 25928 | Wuweizi |
| 102 | Ginsenoside Rh4 | C36H60O8 | 18.72 | 620.4288 | +COOH |  | 665.4259 | | 665.4263 | 0.5 | 15251 | Sanqi |
| 103 | Gomisin N/Kadsuranin | C23H28O6 | 18.73 | 400.1886 | +H |  | 401.1959 | | 401.1961 | 0.5 | 62363 | Wuweizi |
| 104 | Emodin* | C15H10O5 | 18.89 | 270.0528 | -H |  | 269.0456 | | 269.0466 | 3.9 | 715 | Chushizi |
| 105 | Schisantherin A* | C30H32O9 | 19.07 | 536.2046 | +Na | +H | 559.1938 | | 559.1943 | 0.8 | 25928 | Wuweizi |
| 106 | Neokadsuranin | C23H26O7 | 19.21 | 414.1679 | +H |  | 415.1751 | | 415.1753 | 0.4 | 12294 | Wuweizi |
| 107 | Progesterone* | C21H30O2 | 19.82 | 314.2246 | +H |  | 315.2319 | | 315.2313 | -1.8 | 1849 | Ziheche |
| 108 | Linolenic acid | C18H30O2 | 20.21 | 278.2246 | +H |  | 279.2319 | | 279.2319 | 0.3 | 19026 | Tusizi, Chushizi, Chongweizi |
|  |  |  |  |  |  |  |  |  |  |  |  | Mugua, Gouqizi |
| 109 | Tetrahydrocortisone | C21H32O5 | 20.29 | 364.2250 | -H |  | 363.2177 | | 363.2158 | -5.3 | 21579 | Ziheche |
| 110 | Ginsenoside Rg3* | C42H72O13 | 20.29 | 784.4973 | +COOH | +Na,+H | 829.4944 | | 829.4948 | 0.5 | 13208 | Sanqi |
| 111 | Schisandrin A* | C24H32O6 | 20.64 | 416.2199 | +H |  | 417.2272 | | 417.2268 | -0.9 | 26266 | Wuweizi |
| 112 | Schizandrin B* | C23H28O6 | 21.17 | 400.1886 | +H |  | 401.1959 | | 401.1957 | -0.3 | 10217 | Wuweizi |
| 113 | Linoleic acid | C18H32O2 | 24.87 | 280.2402 | -H |  | 279.233 | | 279.2332 | 0.9 | 27082 | Tusizi，Chushizi，Chongweizi |
|  |  |  |  |  |  |  |  |  |  |  |  | Mugua, Gouqizi |

*Compared with reference standards.

**Table S1-4.** Secondary fragment datas within mZJP granules.

| NO. | Compounds | Molecular Formula | tR  (min) | Adduct ion | Theoretical molecular weight  （Da） | Measured molecular weight  (Da) | Error  （ppm） | | MS/MS |
| --- | --- | --- | --- | --- | --- | --- | --- | --- | --- |
| 1 | Argininic acid | C6H14N4O2 | 1.66 | +H | 175.1190 | 175.1188 | -0.9 | 60.0597(11.0),70.0677(100.0),116.0709(10.7),130.0977(8.4),175.1195(30.1) | |
| 2 | Mannose | C6H14O6 | 1.82 | +H | 183.0863 | 183.0858 | -2.7 | 85.0277(33.9),83.0574(33.9),69.0425(33.9),69.0359(100.0) | |
| 3 | Betaine* | C5H12NO2 | 1.82 |  | 118.0868 | 118.08669 | -0.9 | 58.0689(100.0),59.0763(19.7),118.086(54.4) | |
| 4 | Trigonelline | C7H7NO2 | 1.83 | +H | 138.0550 | 138.0550 | 0.2 | 65.0415(7.5),78.0366(12.1),79.0434(8.0),92.0511(33.1),93.0592(7.1),94.0666(25.2),  138.0557(100.0) | |
| 5 | Proline | C5H9NO2 | 1.85 | +H | 116.0706 | 116.0708 | 1.4 | ND | |
| 6 | Quinic acid | C7H12O6 | 1.88 | -H | 191.0561 | 191.0566 | 2.3 | 59.0164(8.4),85.0311(54.6),87.01(9.9),93.036(24.3),109.0305(5.6),127.0407(17.7),  171.0305(5.0),173.0465(8.2),191.0562(100.0) | |
| 7 | Stachydrine hydrochloride* | C7H14NO2+ | 1.9 |  | 144.1025 | 144.1026 | 0.8 | 144.1017(100.0),84.0825(23.7),58.0690(28.4) | |
| 8 | Glucose | C6H12O6 | 1.9 | -H | 179.0561 | 179.0561 | -0.1 | 57.0015(14.4),59.0145(57.2),59.0145(57.2),60.0201(7.2),71.0162(14.4),72.9924(7.2),73.0323(7.2),75.0097(71.2),89.0243(7.2),99.009(14.4),125.0244(7.2),143.0381(21.2),161.0479(7.2),179.0555(100.0) | |
| 9 | D-Mannitol | C6H14O6 | 1.91 | -H | 181.0718 | 181.0721 | 1.6 | ND | |
| 10 | Ascorbyl Glucoside | C12H18O11 | 1.92 | -H | 337.0776 | 337.0779 | 0.9 | 113.996(20.7),115.0043(9.6),127.0039(5.2),157.0144(14.1),174.0173(39.5),175.0256  (5.0),277.0569(52.2),337.0774(100.0) | |
| 11 | β-oxoacteoside | C29H34O16 | 1.92 | -H | 637.1774 | 637.1811 | 5.7 | 179.0564(33.3), 295.0682(100.0),337.0819(5.5),339.0823(5.5),637.1849(7.4) | |
| 12 | Vitamin C | C6H8O6 | 1.94 | +H | 177.0394 | 177.0392 | -0.7 | ND | |
| 13 | Isochlorogenic acid A/B/C | C25H24O12 | 1.97 | -H | 515.1195 | 515.1242 | 9.1 | 516.1196(6.3),111.0447(6.5),263.0758(12.5),101.0272(13.0),179.0552(15.7),341.1093(18.9),515.1593(32.6),173.0447(34.5),191.0551(40.9),173.0088(69.1),515.1264(99.7),111.0092(100.0) | |
| 14 | Shikimic acid | C7H10O5 | 1.98 | -H | 173.0456 | 173.0460 | 2.6 | 173.0081(6.1),111.045(12.1),99.0453(15.1),155.0358(15.1),71.0141(18.2),83.0515  (21.3),137.0244(30.3),73.0302(36.4),173.0442(39.3),93.0354(100.0) | |
| 15 | fumaric acid | C4H4O4 | 2 | -H | 115.0037 | 115.0047 | 8.4 | 71.0146(100.0),115.0011(59.6),115.0075(20.2) | |
| 16 | Malic Acid | C4H6O5 | 2 | -H | 133.0143 | 133.0152 | 7.3 | 71.0154(93.7),72.9944(33.5),89.0245(10.4),115.0039(100.0),133.0143(69.5) | |
| 17 | Hypoxanthine* | C5H4N4O | 2.02 | +H | 137.0458 | 137.0459 | 0.5 | 137.0467(100.0),119.0354(49.7),110.0341(25.1),95.0875(12.6),94.0410(25.1),82.0411(25.1),81.0758(12.6),65.0161(12.6),55.0385(25.1),55.0328(12.6) | |
| 18 | (Z)-Aconitic Acid | C6H6O6 | 2.04 | -H | 173.0092 | 173.0098 | 3.5 | 57.0359(16.8),67.018(33.6),85.0296(33.6),111.0081(100.0),129.0176(16.8),155.0011  (16.8),173.0117(33.6) | |
| 19 | Citric Acid | C6H8O7 | 2.06 | -H | 191.0197 | 191.0204 | 3.5 | 57.037(11.4),67.0205(13.6),85.0307(35.1),87.0097(60.8),111.0089(100.0),129.0194  (10.3),173.0092(5.9),191.0198(30.2) | |
| 20 | Uridine | C9H12N2O6 | 2.63 | -H | 243.0623 | 243.0625 | 0.8 | 153.0293(33.6),122.0262(16.8),152.0337(49.6),243.062(83.2),110.0238(100.0) | |
| 21 | Guanosine* | C10H13N5O5 | 2.67 | -H | 282.0844 | 282.0844 | 0.1 | 80.0276(12.5),108.0207(16.7),133.0149(41.9), 150.0418(100.0),282.0845(50.3) | |
| 22 | Inosine* | C10H12N4O5 | 2.68 | -H | 267.0735 | 267.0738 | 1 | 92.0266(19.8),108.0193(10.0),135.0317(100.0),266.2622(5.0),267.0726(59.7) | |
| 23 | l-isoleucine | C6H13NO2 | 2.78 | +H | 132.1019 | 132.1020 | 0.8 | 69.0756(5.4),86.0985(100.0),132.1026(16.0) | |
| 24 | Succinic acid* | C4H6O4 | 2.82 | -H | 117.0193 | 117.0204 | 8.7 | 55.0216(10.5),73.0307(100.0),99.0094(10.5),117.0199(20.2) | |
| 25 | D-tert-Leucine | C6H13NO2 | 2.99 | +H | 132.1019 | 132.1020 | 1 | 69.0756(5.4),86.0985(100.0),132.1026(16.0) | |
| 26 | Gallic acid | C7H6O5 | 3.25 | -H | 169.0143 | 169.0149 | 3.6 | 81.0338(6.6),97.0316(6.6),124.0168(13.4),125.0245(100.0),169.0146(26.3) | |
| 27 | Sesaminol | C20H18O7 | 3.91 | -H | 369.0980 | 369.1017 | 10 | 191.0561(7.2),180.0585(14.4),113.0261(21.2),119.0353(28.4),89.0267(35.6),369.104  (50.0),144.0462(85.6),145.0479(100.0) | |
| 28 | phenylalanine | C9H11NO2 | 3.91 | -H | 164.0717 | 164.0723 | 3.4 | 72.0095(11.7),103.0563(39.7),147.0453(100.0),164.0717(55.0) | |
| 29 | [Nitidine](https://pubchem.ncbi.nlm.nih.gov/compound/4501) | C21H18NO4+ | 4.34 |  | 348.1236 | 348.1287 | 14.6 | 294.1917(33.9),293.1831(67.7),169.0504(100) | |
| 30 | Geniposidic acid* | C16H22O10 | 4.46 | -H | 373.1140 | 373.1141 | 0.2 | 121.0658(10.5),123.0456(100.0),149.0607(64.6),167.0711(34.5),193.0498(7.0),  211.0606(42.2),373.1139(80.5) | |
| 31 | Neochlorogenic acid | C16H18O9 | 5.28 | -H | 353.0878 | 353.0882 | 1.2 | 191.0555(100.0),353.0863(6.6) | |
| 32 | protocatechuic acid | C7H6O4 | 5.35 | -H | 153.0193 | 153.0201 | 5.1 | 65.0048(5.5),91.0198(7.0),108.023(12.7),109.0294(100.0),153.0194(31.8) | |
| 33 | Aucubin | C15H22O9 | 6.18 | -H | 345.1191 | 345.1194 | 0.7 | 179.0559(11.2),89.0231(22.5),345.1216(33.2),299.1143(100.0) | |
| 34 | Quercetin 3-O-robinobioside | C27H30O17 | 6.65 | -H | 625.1410 | 625.1419 | 1.3 | 299.0192(31.2),301.0354(19.4),462.0808(29.8),463.0893(23.7),625.1442(100.0) | |
| 35 | Catechin | C15H14O6 | 6.68 | -H | 289.0718 | 289.0721 | 1.1 | 159.0446(10.7),179.0345(11.1),161.0605(12.6),187.0397(12.6),137.0244(16.4),  151.0395(17.5),125.0244(21.9),205.0503(23.7),109.0297(27.1),123.0454(31.5),  203.0712(35.8),245.0822(43.5),289.0719(100.0) | |
| 36 | Cryptochlorogenic acid* | C16H18O9 | 6.78 | -H | 353.0878 | 353.0882 | 1.2 | 191.0555(100.0),353.0863(6.6) | |
| 37 | Chlorogenic acid* | C16H18O9 | 6.88 | -H | 353.0878 | 353.0881 | 1 | 353.0886(32.4),191.0560(100.0),179.0345(43.1),173.0453(69.7),135.0447(50.5) | |
| 38 | 4-Hydroxybenzoic acid | C7H6O3 | 6.91 | -H | 137.0244 | 137.0252 | 5.3 | 108.0229(9.7),136.0171(19.5),137.0245(100.0) | |
| 39 | Quercetin-3-O-α-L-rhamnopyranosyl-(1→6)-β-D-galactopyranoside | C27H30O16 | 7.1 | -H | 609.1461 | 609.1458 | -0.5 | 300.0274(40.8),301.0352(29.4),609.1477(100.0) | |
| 40 | Epicatechin* | C15H14O6 | 7.4 | -H | 289.0718 | 289.0719 | 0.5 | 123.0454(31.5),125.0244(21.9),135.0462(6.6),137.0244(16.4),151.0395(17.5),  159.0446(10.7),161.0605(12.6),179.0345(11.1),187.0397(12.6),203.0712(35.8),  205.0503(23.7),221.0819(8.5), 245.0822(43.5),289.0719(100.0) | |
| 41 | Caffeic acid* | C9H8O4 | 7.48 | -H | 179.0350 | 179.0356 | 3.3 | 134.0372(27.2),135.0454(100.0),179.0347(18.5) | |
| 42 | Phenylacetic acid | C8H8O2 | 7.49 | -H | 135.0452 | 135.0460 | 5.9 | 107.0534(18.3),117.0369(18.3),134.0385(63.8),135.0461(100.0) | |
| 43 | Daphnetin/Esculetin | C9H6O4 | 7.51 | -H | 177.0193 | 177.0199 | 3.1 | ND | |
| 44 | 8-epi-Loganic acid | C16H24O10 | 7.55 | -H | 375.1297 | 375.1309 | 3.4 | 375.1320(37.5),255.0877 (100.0),241.0744(12.6),212.0824(24.9) | |
| 45 | Riboflavin | C17H20N4O6 | 7.55 | +H | 377.1456 | 377.1459 | 0.9 | 172.0866(27.4),198.0660(19.6),243.0870(59.0),359.1382(6.3),377.1465(100.0) | |
| 46 | Leonurine | C14H21N3O5 | 7.9 | +H | 312.1554 | 312.1551 | -0.9 | 114.1028(33.2),138.0301(16.8),153.0542(24.8),181.0496(100.0), 312.1544(91.6) | |
| 47 | benzoic acid | C7H6O2 | 8.35 | -H | 121.0295 | 121.0304 | 7.1 | 91.0202(7.4),92.028(52.7),93.0349(5.8),120.0225(13.2),121.03(100.0) | |
| 48 | Quercetin-3-O-apose-(1→2)-galactoside | C26H28O16 | 8.45 | -H | 595.1305 | 595.1299 | -0.9 | 255.0303(6.0),271.0246(11.3),300.0271(90.6),301.0349(23.1),595.1311(100.0) | |
| 49 | 4-Hydroxycinnamic acid/  cis-Hydroxycinnamic acid | C9H8O3 | 8.63 | -H | 163.0401 | 163.0409 | 4.8 | 93.0344(14.6),117.0354(8.6),119.0502(100.0),163.039(18.7) | |
| 50 | Naringenin-7-O-glucoside | C21H22O10 | 8.76 | -H | 433.1140 | 433.1143 | 0.5 | 133.0298(23.0),135.0465(8.4),161.0249(78.0), 323.0773(6.2),433.1144(100.0) | |
| 51 | Phenylacetaldehyde | C8H8O | 9.01 | -H | 119.0502 | 119.0513 | 8.9 | 93.0360(18.8),117.0368(9.5),119.0504(100.0) | |
| 52 | 5-Hydroxycinnamic acid/cis-Hydroxycinnamic acid | C9H8O3 | 9.01 | -H | 163.0401 | 163.0409 | 4.8 | 93.0344(14.6),117.0354(8.6),119.0502(100.0),163.0390(18.7) | |
| 53 | Quercetin 3-O-robinobioside | C27H30O16 | 9.05 | +H | 611.1607 | 611.1613 | 1 | 303.0499(100.0),465.1044(9.4) | |
| 54 | Verbascoside* | C29H36O15 | 9.16 | -H | 625.2127 | 625.2131 | 0.7 | 161.0242(45.7),461.1662(16.4),623.1978(100.0),624.2038(11.5) | |
| 55 | Rutin | C27H30O16 | 9.17 | -H | 609.1461 | 609.1467 | 1 | 300.0274(40.8),301.0352(29.4),609.1477(100.0) | |
| 56 | Hyperoside* | C21H20O12 | 9.45 | -H | 463.0882 | 463.0883 | 0.3 | 255.0309(18),271.0257(33.2),300.0284(100.0),301.0365(48.2),463.0886(70.0) | |
| 57 | Plantamajoside/Plantainoside D | C29H36O16 | 9.46 | -H | 639.1931 | 639.1926 | -0.7 | 161.0249(29.3),477.1647(9.2),639.196(100.0) | |
| 58 | Martynoside | C31H40O15 | 9.61 | -H | 651.2294 | 651.2301 | 1 | 151.04(20.4),339.1245(11.9),357.135(64.9),651.2312(100.0),652.2353(11.9) | |
| 59 | Isoquercitrin | C21H20O12 | 9.64 | -H | 463.0882 | 463.0883 | 0.3 | 255.0309(18.0),271.0257(33.2),300.0284(100.0),301.0365(48.2),463.0886(70.0) | |
| 60 | Scopoletin | C10H8O4 | 9.69 | +H | 193.0495 | 193.0494 | -0.5 | 66.0482(10.2),77.0398(8.2),81.03612(10.2),94.04445(15.3),122.0376(19.4),133.0297  (44.9),137.0611(12.2), 150.0306(21.4),178.0263(33.7),193.0497(100.0) | |
| 61 | Ferulic acid* | C10H10O4 | 9.72 | -H | 193.0506 | 193.0514 | 3.8 | 133.0302(39.6),134.0382(100.0),149.0607(14.9),178.0275(24.5),193.0517(34.4) | |
| 62 | Heteroclitin G | C22H24O7 | 9.76 | +H | 401.1595 | 401.1579 | -4.1 | 401.1579(100.0) | |
| 63 | Forsythoside B | C34H44O19 | 9.79 | -H | 755.2404 | 755.2410 | 0.8 | 161.0256(12.2),593.2116(9.8),709.2642(5.2),755.2458(100.0),756.2509(17.2) | |
| 64 | Calceolarioside A/B | C23H26O11 | 10 | +H | 479.1548 | 479.1549 | 0.2 | ND | |
| 65 | Isoacteoside* | C29H36O15 | 10.14 | -H | 623.1981 | 623.1974 | -1.2 | 161.0242(45.7),461.1662(16.4),623.1978(100.0),624.2038(11.5) | |
| 66 | Methyl sinapate | C12H14O5 | 10.33 | +H | 239.0914 | 239.0896 | -7.4 | 221.0859(11.2),239.0894(100.0) | |
| 67 | Kaempferol | C15H10O6 | 11.04 | +H | 287.0550 | 287.0548 | -0.7 | 259.0613(5.5),213.0624(11),258.0470(11.3),121.0284(16.2),287.0538(100.0) | |
| 68 | Esculetin | C9H6O4 | 11.1 | -H | 177.0193 | 177.0198 | 2.7 | ND | |
| 69 | Astragalin/ Kaempferol-3-O-galactoside | C21H20O11 | 11.2 | -H | 447.0933 | 447.0933 | 0 | 227.0344(33.2),255.0293(58.4),284.0329(66.1),285.0409(32.7),447.0947(100.0) | |
| 70 | Physcion diglucoside | C28H32O15 | 11.37 | -H | 607.1668 | 607.1671 | 0.4 | 284.0328(26),299.0557(100.0),607.1655(38.7) | |
| 71 | KaeMpferol 7-O-rhaMnoside | C21H20O10 | 11.51 | +COOH | 477.1028 | 477.1033 | 1.2 | 243.0309(18.5),257.0467(7.7),271.0252(18.3),285.041(19.3),286.0487(6.9),299.0201  (9.4),300.0302(5.2),314.0439(51.8),315.0514(11.2),357.0597(5.6),477.1051(100.0) | |
| 72 | Nepitrin | C22H22O12 | 11.51 | -H | 477.1039 | 477.1034 | -1 | 243.0309(18.5),257.0467(7.7),271.0252(18.3),285.041(19.3),286.0487(6.9),299.0201  (9.4),300.0302(5.2),314.0439(51.8),315.0514(11.2),357.0597(5.6),477.1051(100.0) | |
| 73 | Apigenin | C15H10O5 | 11.95 | -H | 269.0456 | 269.0474 | 6.7 | 117.0363(16.8),149.0231(16.8),159.0431(16.8),225.0583(33.6),269.0452(100.0) | |
| 74 | Azelaic acid | C9H16O4 | 12.37 | -H | 187.0976 | 187.0981 | 2.9 | 97.0671(11.9),123.0814(15.5),125.0974(100.0),169.0872(14.3),187.0974(72.8) | |
| 75 | Cuscutoside D | C37H46O21 | 12.56 | +Na | 849.2424 | 849.2426 | 0.3 | 849.4056(8.7),849.4624(8.7),849.2412(100.0) | |
| 76 | Notoginsenoside R1* | C47H80O18 | 12.78 | +COOH | 977.5316 | 977.5325 | 1 | 799.4852(7.8),931.5261(100.0),932.5291(23.7),977.5326(16.2),978.5362(7.4) | |
| 77 | Notoginsenoside Re* | C48H82O18 | 13.19 | +COOH | 991.5472 | 991.5482 | 0.9 | 945.5447(100.0),946.5483(38.1),991.5519(20.8),992.5551(10.1) | |
| 78 | Ginsenoside Mc | C41H70O12 | 13.31 | +COOH | 799.4838 | 799.4842 | 0.5 | 161.0458(10.6),475.3794(8.5),637.4338(31.9), 799.4885(100.0),800.487(10.6) | |
| 79 | Notoginsenoside Rf* | C42H72O14 | 13.32 | +COOH | 845.4893 | 845.4901 | 1 | 161.0459(16.8),179.0561(10.0),475.3791(15.3),637.431(42.4),638.4382(7.0),799.4841(83),800.4874(11.9),845.4897(100.0),846.0734(6.5),846.4917(70.1) | |
| 80 | Tiliroside | C30H26O13 | 13.41 | -H | 593.1301 | 593.1295 | -1 | 255.0293(9.9),256.0383(6.2),284.0331(34),285.0409(51.4),593.1305(100) | |
| 81 | Quercetin* | C15H10O7 | 13.47 | -H | 301.0354 | 301.0357 | 0.9 | 302.041(7.9),121.0307(10.2),178.9989(12.8),151.0038(65.2),301.0348(100) | |
| 82 | Cuscutoside C | C26H28O12 | 14.4 | +Na | 555.1473 | 555.1463 | -1.9 | 203.0508(16.8),555.1148(16.8),555.1501(100.0) | |
| 83 | Luteolin* | C15H10O6 | 14.43 | -H | 285.0405 | 285.0409 | 1.6 | 286.0445(5.2),285.0416(100.0) | |
| 84 | Isorhamnetin | C16H12O7 | 14.65 | -H | 315.0510 | 315.0513 | 0.8 | 316.0594(11),301.0382(11.3),148.017(16.2),163.0035(16.2),247.0956(21.7),151.0048  (32.7),315.052(88.5),300.0283(100) | |
| 85 | Ginsenoside Rg1* | C42H72O14 | 15.02 | +COOH | 845.4893 | 845.4897 | 0.5 | 119.0355(8.9),161.0459(16.8),179.0561(10),475.3791(15.3),637.431(42.4),638.4382  (7),799.4841(83),800.4874(11.9),845.4897(100),846.0734(6.5),846.4917(70.1) | |
| 86 | Notoginsenoside R2/  Ginsenoside F5 | C41H70O13 | 15.29 | +COOH | 815.4788 | 815.4794 | 0.8 | 475.38(12.3),637.4329(14.3),769.4758(100),770.4798(19.7),815.482(18.5) | |
| 87 | Ginsenoside Rg2* | C42H72O13 | 15.58 | +COOH | 829.4944 | 829.4950 | 0.8 | 637.4352(8.5),783.4926(100),784.4955(25.3),829.4986(27.1),830.5032(7.1) | |
| 88 | Ginsenoside F1* | C36H62O9 | 15.83 | +COOH | 683.4365 | 683.4369 | 0.6 | 637.4335(12.1),683.4386(100),684.4451(10.6) | |
| 89 | Cuscuta acid C | C38H68O20 | 15.94 | -H | 843.4231 | 843.4226 | -0.6 | 117.0562(23.3),163.0614(17.5),237.0988(8.6),243.1966(100),244.2008(7.9),263.1147  (6.3),281.1245(22),333.1191(5.5),351.1296(11.7),407.2645(12),633.3498(21.9),  635.3642(14.6),659.3654(8.8),677.374(7.8), 843.4248(59.9),844.4271(16.7) | |
| 90 | Ginsenoside Rb1* | C54H92O23 | 16.11 | +COOH | 1153.6001 | 1153.5990 | -1 | 1107.598(100),1108.099(9.4),1108.603(57),1153.603(11.8) | |
| 91 | Wogonin/Genkwanin | C16H12O5 | 16.44 | -H | 283.0612 | 283.0613 | 0.3 | 110.0036(9.7),163.0047(16.2),165.9906(12.9),196.0502(12.9),267.0251(9.8),268.0375(100),269.0378(6.5),283.0615(29.1) | |
| 92 | Ginsenoside Rh1* | C36H62O9 | 16.66 | +COOH | 683.4365 | 683.4369 | 0.6 | 637.4335(12.1),683.4386(100),684.4451(10.6) | |
| 93 | Wogonin/Genkwanin | C16H12O5 | 16.86 | -H | 283.0612 | 283.0614 | 0.8 | 110.0036(9.7),163.0047(16.2),165.9906(12.9),196.0502(12.9),267.0251(9.8),268.0375(100),269.0378(6.5),283.0615(29.1) | |
| 94 | Veraguensin | C22H28O5 | 16.89 | +H | 373.2010 | 373.2004 | -1.5 | 266.9945(9.7),268.9976(19.1),355.1866(19.1),357.0681(28.7),373.1989(100) | |
| 95 | Schisandrin* | C24H32O7 | 16.94 | +H | 433.2221 | 433.2217 | -1 | 331.1177(9.5),332.1263(6.5),338.1506(12.6),342.1474(16.8),345.1359(5.2),346.1412  (22),347.151(5.1),353.1763(10.3),354.1463(5.1),359.1512(6.5),369.1699(17.2),  373.1658(5.8),384.194(63.4),400.1882(16.9),415.2123(100),433.2247(6.6) | |
| 96 | Gomisin J | C22H28O6 | 17.12 | +H | 389.1959 | 389.1961 | 0.7 | 372.102(11.2),356.0731(22.5),357.1728(33.2),287.0912(44.4),389.1985(100) | |
| 97 | Ginsenoside RG5 | C42H70O12 | 17.55 | +H | 767.4940 | 767.4943 | 0.4 | 749.5066(10),369.3147(10.1),587.4271(13.3),207.1738(16.7),605.4481(20.1),  325.1139(23.4),443.3872(26.8),767.4922(30),425.3781(70.8),407.3665(100) | |
| 98 | Schisandrol B* | C23H28O7 | 17.63 | +Na | 439.1727 | 439.1732 | 1 | 439.1737(100.0) | |
| 99 | Ginsenoside Rd* | C48H82O18 | 17.67 | +COOH | 991.5472 | 991.5467 | -0.5 | 945.5447(100),946.5483(38.1),991.5519(20.8),992.5551(10.1) | |
| 100 | Angeloylgomisin H | C28H36O8 | 18.18 | +H | 501.2483 | 501.2478 | -1 | 409.1987(7),359.1518(9.9),386.1692(9.9),318.1122(13.3),345.1385(16.6),355.1564  (16.6),332.1267(20),369.1709(20),370.1788(26.7),483.2383(26.7),401.1975(100) | |
| 101 | Gomisin G | C30H32O9 | 18.34 | +H | 537.2119 | 537.2098 | -4 | 340.1292(22.5),341.1368(9.4),342.1079(16.8),343.1177(5.6),343.1551(7.4),356.1255  (5.6),366.1467(5.6),367.1559(5.6),371.1509(28.1),373.1283(15),385.1645(9.3),  400.1529(5.6),415.1762(26.6),437.1571(23),520.1346(5.8),537.2087(100) | |
| 102 | Ginsenoside Rh4 | C36H60O8 | 18.72 | +COOH | 665.4259 | 665.4263 | 0.5 | 619.4252(11.5),666.4322(11.5),665.4281(100.0) | |
| 103 | Gomisin N/Kadsuranin | C23H28O6 | 18.73 | +H | 401.1959 | 401.1961 | 0.5 | 345.1325(6.1),359.1498(7.7),386.1721(10.7),355.1554(12.3),313.1074(13.8),332.1261(15.3),369.1709(17.1),370.1775(30.7),401.1973(100) | |
| 104 | Emodin* | C15H10O5 | 18.89 | -H | 269.0456 | 269.0466 | 3.9 | 225.0583(33.6),269.0452(100.0) | |
| 105 | Schisantherin* | C30H32O9 | 19.07 | +Na | 559.1938 | 559.1943 | 0.8 | 559.1965(100.0),437.1588(26.7),415.1755(32.1),371.1491(40.1),342.1100 (16.0),340.1328 (37.7) | |
| 106 | Neokadsuranin | C23H26O7 | 19.21 | +H | 415.1751 | 415.1753 | 0.4 | 342.1107(19.5),367.1553(19.5),373.1317(19.5),119.0864(26.1),325.1058(26.1),  340.1295(32.7),371.1509(32.7),415.1705(100) | |
| 107 | Progesterone* | C21H30O2 | 19.82 | +H | 315.2319 | 315.2313 | -1.8 | 97.0627(100),109.0643(50),315.1959(50),315.2268(100),315.339(50) | |
| 108 | Linolenic acid | C18H30O2 | 20.21 | +H | 279.2319 | 279.2319 | 0.3 | 121.0299(7.7),149.0242(100.0) | |
| 109 | Tetrahydrocortisone | C21H32O5 | 20.29 | -H | 363.2177 | 363.2158 | -5.3 | 171.1016(19.7),277.2164(46.3),295.228(100),296.2316(6.7),317.2063(19.7),344.9438  (13.3),363.2148(72.7),364.2129(6.7) | |
| 110 | Ginsenoside Rg3* | C42H72O13 | 20.29 | +COOH | 829.4944 | 829.4948 | 0.5 | 637.4352(8.5),783.4926(100),784.4955(25.3),829.4986(27.1),830.5032(7.1) | |
| 111 | Schisandrin A* | C24H32O6 | 20.64 | +H | 417.2272 | 417.2268 | -0.9 | 386.2136(6.6),270.0863(9.7),273.1109(16.3),347.1488(19.5),315.1243(19.8),285.1133(22.8),402.2047(23.1),301.11(32.5),417.2268(81.6),316.1291(100) | |
| 112 | Schizandrin B* | C23H28O6 | 21.17 | +H | 401.1959 | 401.1957 | -0.3 | 386.1721(10.7),355.1554(12.3),313.1074(13.8),332.1261(15.3),369.1709(17.1),  370.1775(30.7),401.1973(100) | |
| 113 | Linoleic acid | C18H32O2 | 24.87 | -H | 279.2330 | 279.2332 | 0.9 | 279.2339(100.0),280.2347(6.5) | |

*Compared with reference standard

**Table S1-5.** Contents of the main components detected in the mZJP granules（n=3, quantitative analysis by LC-MS/MS）

| NO. | Compounds | Determined conc.  (ng/mL) | Dilution ratio | Content of crud herbs  （μg/g） | Source |
| --- | --- | --- | --- | --- | --- |
| 1 | Betaine | 315.3±8.3 | 300 | 1419±37 | Gouqizi |
| 2 | Stachydrine hydrochloride | 181.7±5.7 | 300 | 817.7± 25.7 | Chongweizi |
| 3 | Succinic acid | 53.80 ±1.50 | 300 | 242.1±6.8 | Mugua |
| 4 | Chlorogenic acid | 73.20± 2.23 | 300 | 329.4±10.0 | Tusizi, Mugua,  Gouqizi |
| 5 | Acteoside | 8.047 ±0.258 | 300 | 36.21 ±1.16 | Cheqianzi,  Chongweizi |
| 6 | Rutin | 6.233 ±0.170 | 300 | 28.05 ±0.77 | Cheqianzi, Chongweizi, Mugua, Gouqizi |
| 7 | Hyperoside | 39.77±0.86 | 300 | 179.0 ±3.9 | Tusizi, Mugua, Gouqizi |
| 8 | Isoacteoside | 16.47±2.71 | 300 | 74.12 ±12.20 | Cheqianzi, Chongweizi |
| 9 | Ginsenoside Re | 98.77±16.75 | 100 | 148.2±25.1 | Sanqi |
| 10 | Ginsenoside Rg1 | 300.7±18.1 | 300 | 1353 ±81 | Sanqi |
| 11 | Ginsenoside Rg2 | 20.37±1.15 | 300 | 91.67 ±5.18 | Sanqi |
| 12 | Schisandrin | 5.537±0.150 | 300 | 24.92 ±0.68 | Wuweizi |
| 13 | Ginsenoside Rd | 34.80± 4.35 | 300 | 156.6±19.58 | Sanqi |
| 14 | Progesterone | 0.05973± 0.00038 | 100 | 0.08960± 0.00057 | Ziheche |
| 15 | Schisandrin A | 0.08937 ±0.00117 | 100 | 0.1341± 0.0018 | Wuweizi |

**Figure S1-1.**


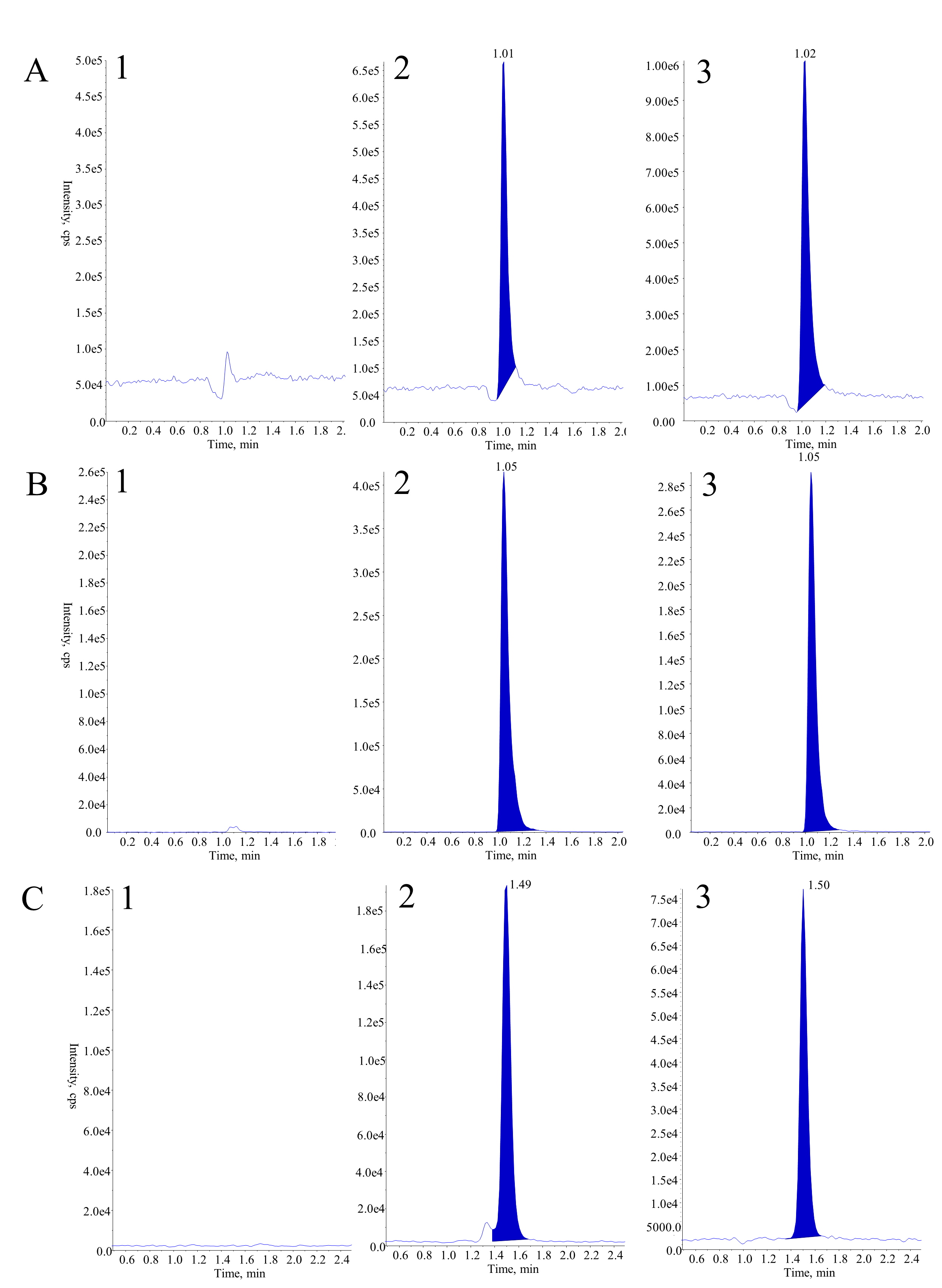

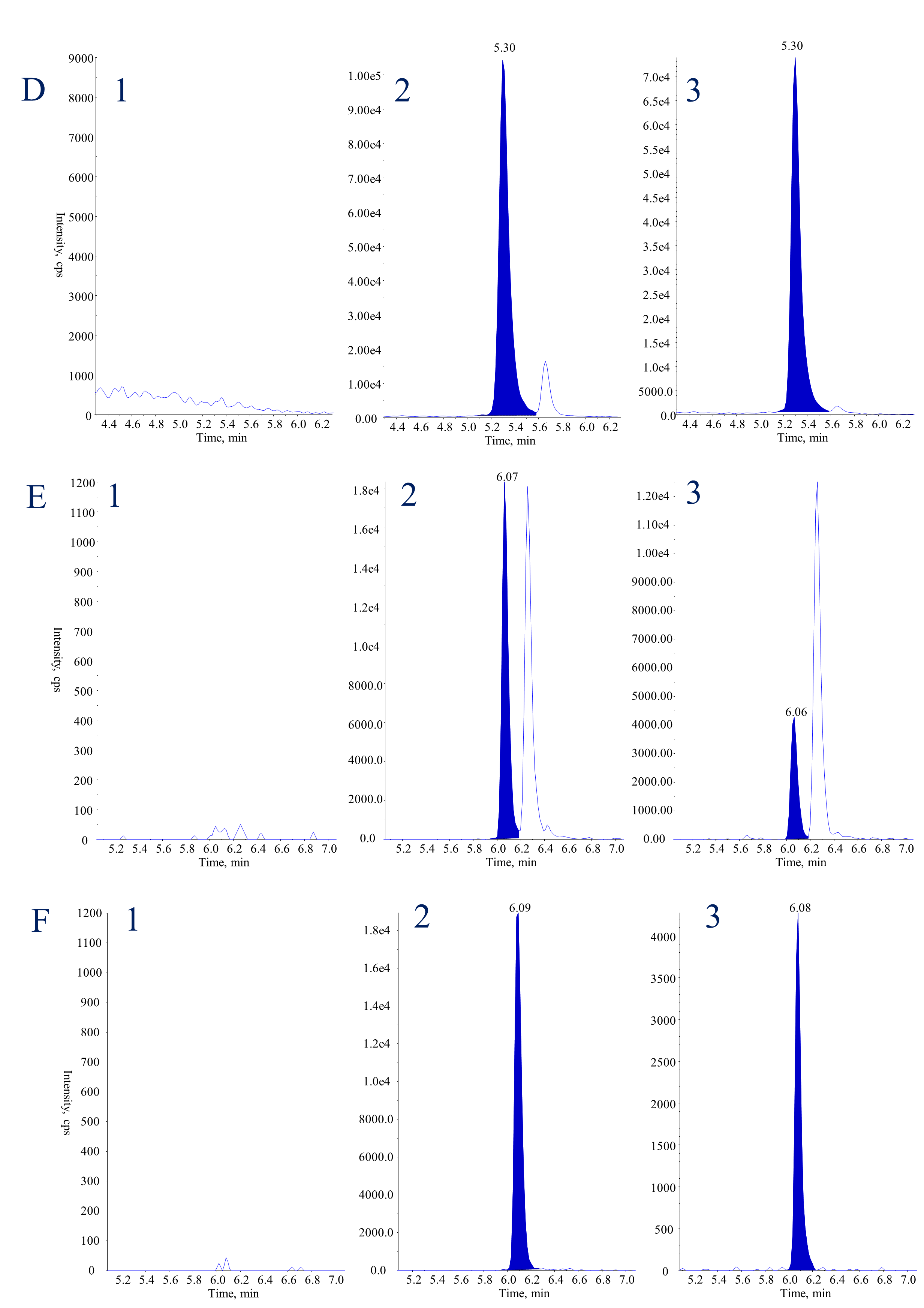


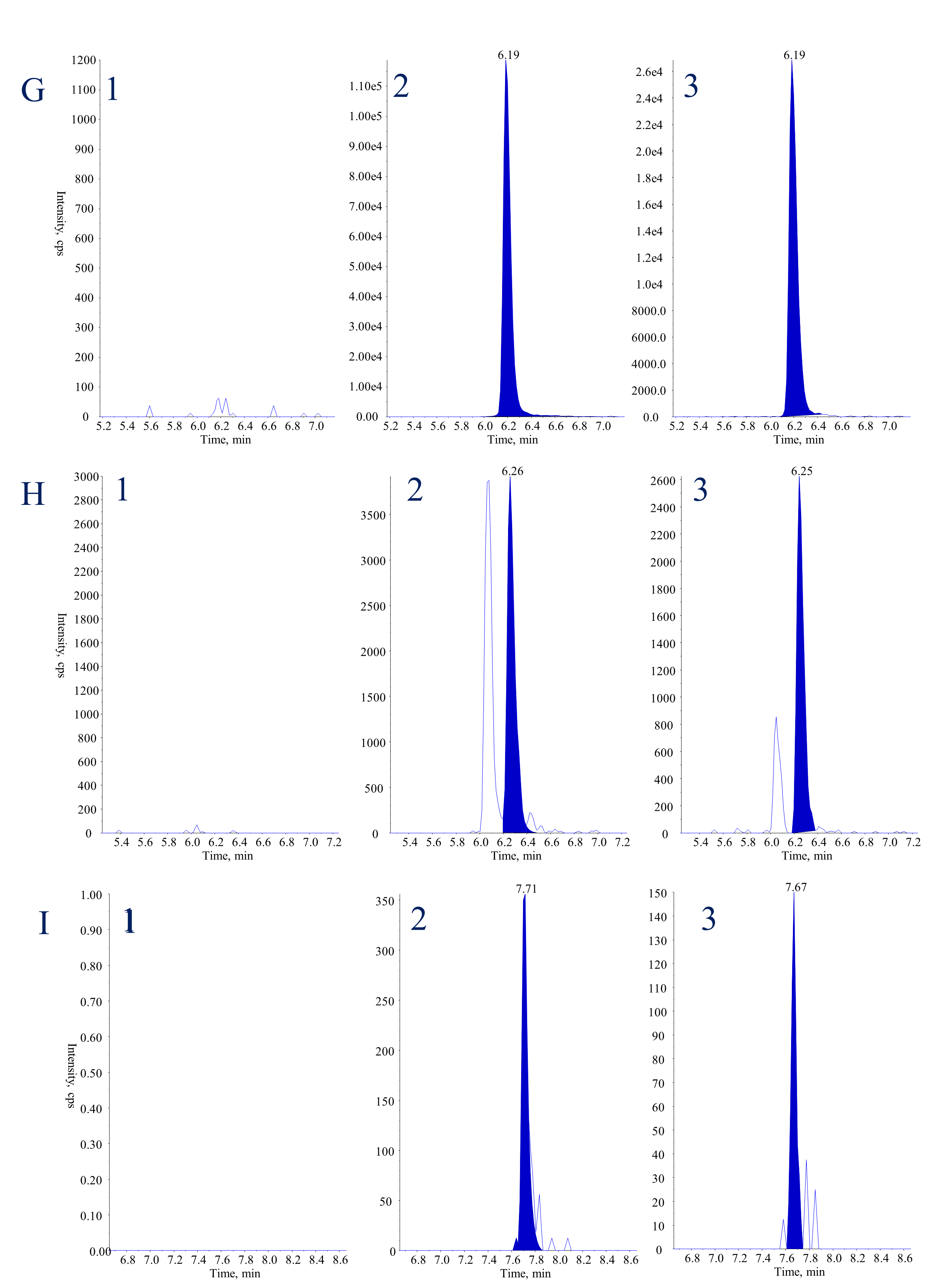

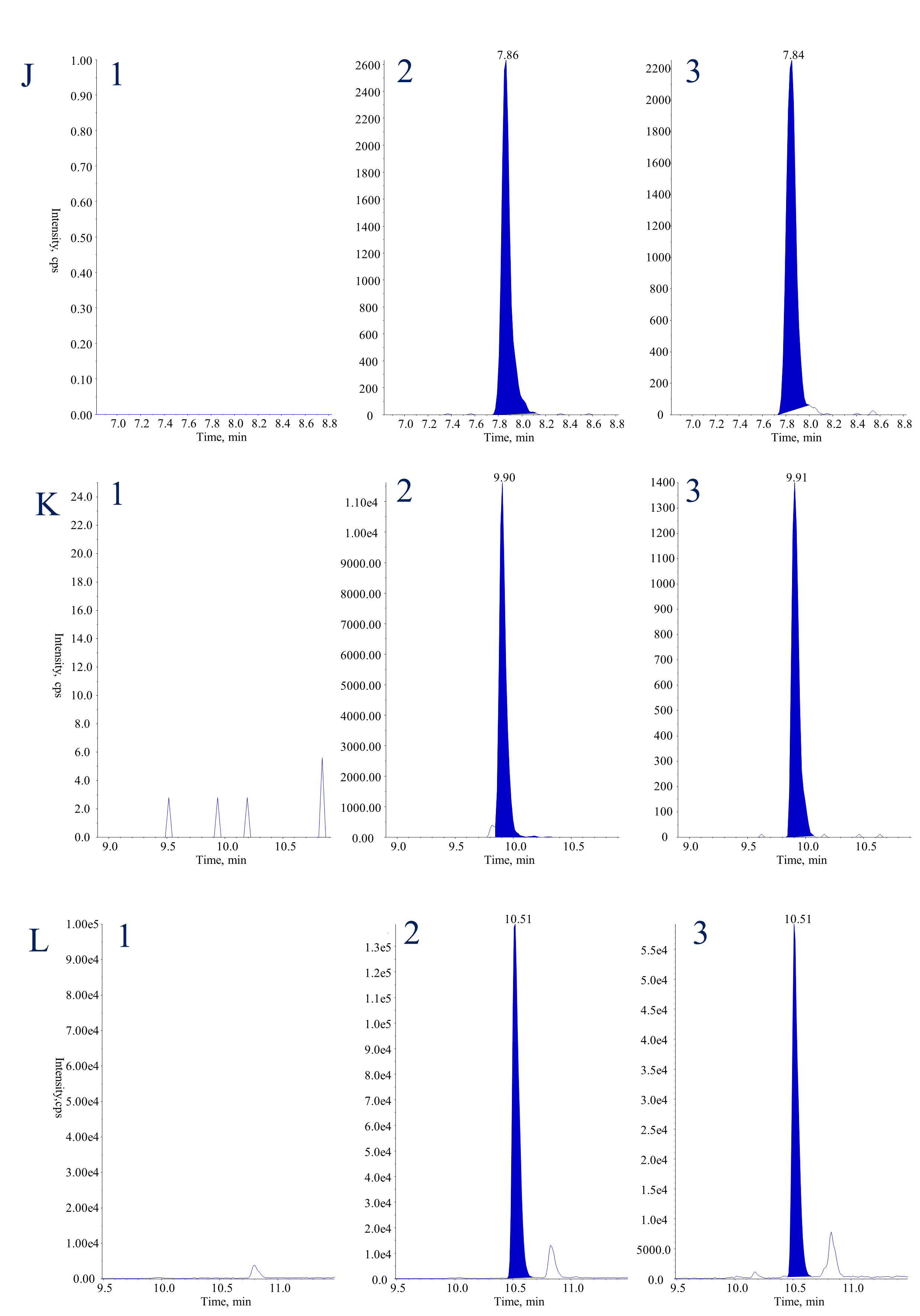


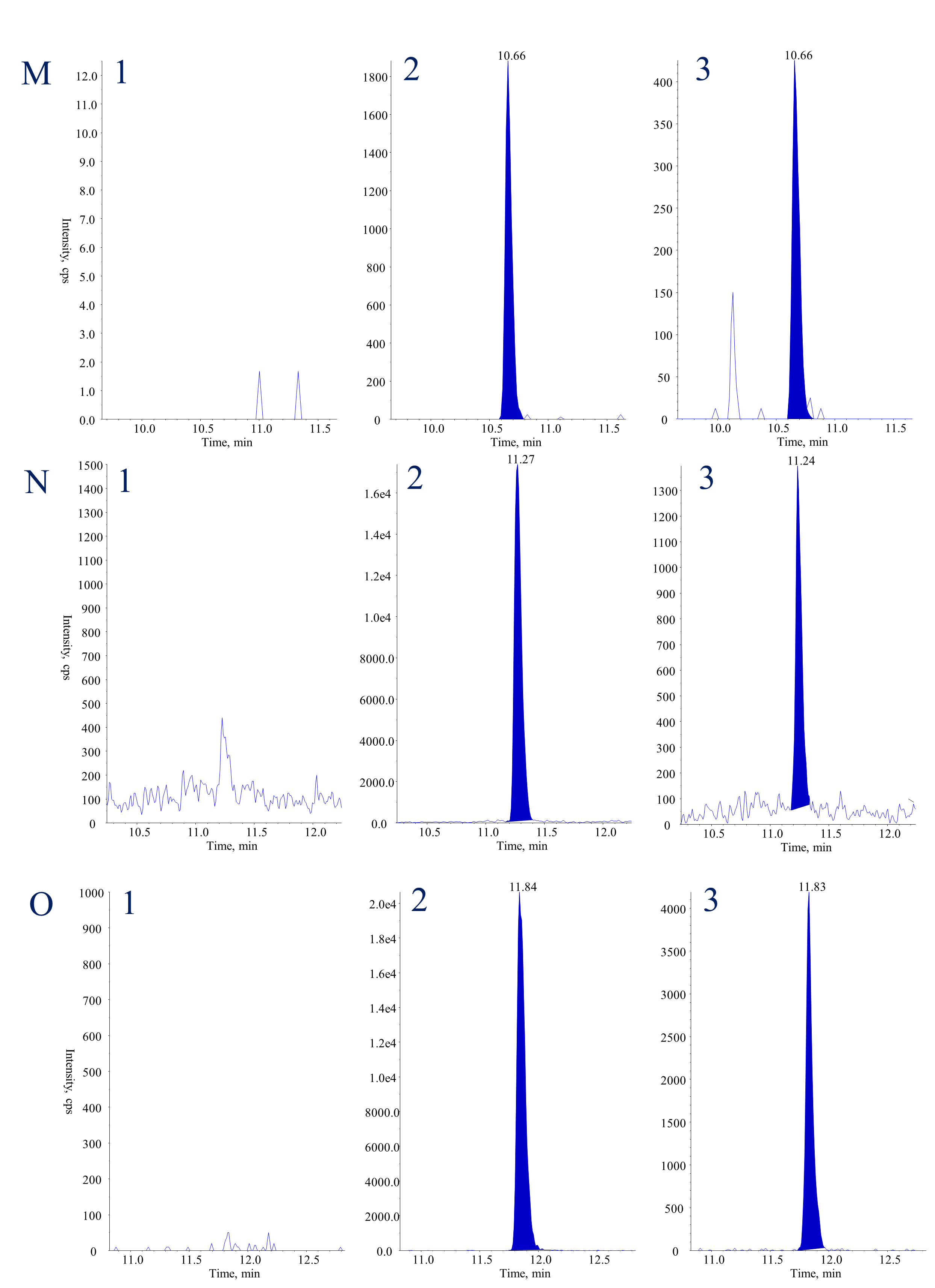

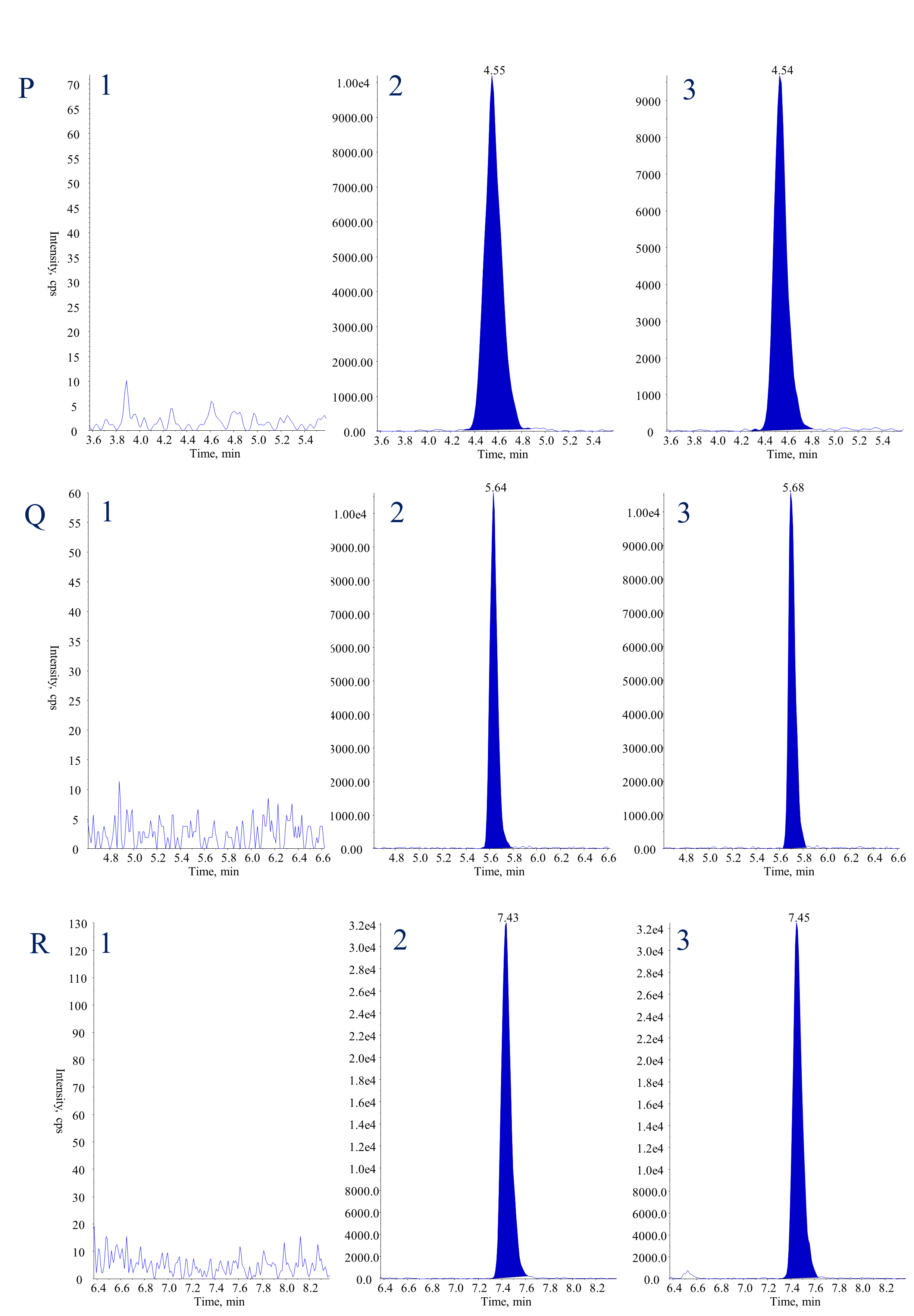


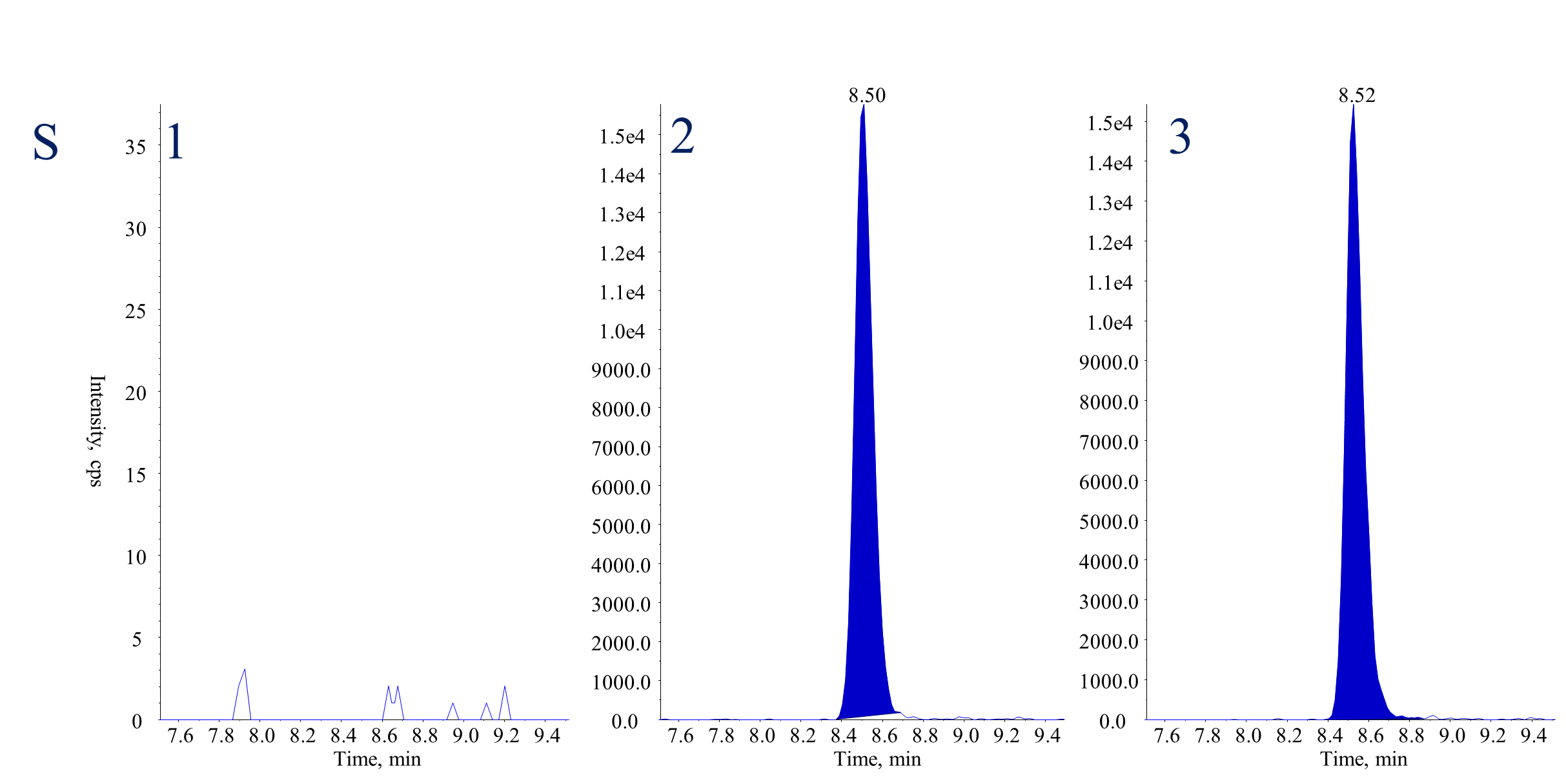


Figure S1. LC-MS/MS chromatograms of the mZJP(granules) components（1. Blank solvent; 2. Control；3. mZJP granules samples; A. Betaine; B. Stachydrine; C. Succinic Acid; D. Chlorogenic Acid; E. Acteoside; F. Rutin; G. Hyperoside; H. Isoacteoside; I. Ginsenoside Re; J. Ginsenoside Rg1; K. Ginsenoside Rg2; L. Schisandrin; M. Ginsenoside Rd; N. Progesterone;
O. Schisantherin A; P. Theophylline; Q. Tinidazole; R. Phenacetin; S. Chlorzoxazone)
